# Shifts in the spatiotemporal profile of inflammatory phenotypes of innate immune cells in the rat brain following acute intoxication with the organophosphate diisopropylfluorophosphate

**DOI:** 10.1186/s12974-024-03272-8

**Published:** 2024-11-04

**Authors:** Peter M. Andrew, Jeremy A. MacMahon, Pedro N. Bernardino, Yi-Hua Tsai, Brad A. Hobson, Valerie A. Porter, Sydney L. Huddleston, Audrey S. Luo, Donald A. Bruun, Naomi H. Saito, Danielle J. Harvey, Amy Brooks-Kayal, Abhijit J. Chaudhari, Pamela J. Lein

**Affiliations:** 1 Department of Molecular Biosciences, Davis, School of Veterinary Medicine, University of California, Davis, CA 95616 USA; 2 Center for Molecular and Genomic Imaging, College of Engineering, University of California, DavisDavis, CA 95616 USA; 3 Department of Biomedical Engineering, College of Engineering, University of California, DavisDavis, CA 95616 USA; 4 Department of Public Health Sciences, Davis, School of Medicine, University of California, Davis, CA 95616 USA; 5 Department of Neurology, Davis, School of Medicine, University of California, Sacramento, CA 95817 USA; 6 Department of Radiology, Davis, School of Medicine, University of California, Sacramento, CA 95817 USA

**Keywords:** Astrocytes, Microglia, Nerve agent, Pesticide, Seizures, Status epilepticus

## Abstract

Acute intoxication with cholinesterase inhibiting organophosphates (OP) can produce life-threatening cholinergic crisis and status epilepticus (SE). Survivors often develop long-term neurological consequences, including spontaneous recurrent seizures (SRS) and impaired cognition. Numerous studies implicate OP-induced neuroinflammation as a pathogenic mechanism contributing to these chronic sequelae; however, little is known about the inflammatory phenotype of innate immune cells in the brain following acute OP intoxication. Thus, the aim of this study was to characterize the natural history of microglial and astrocytic inflammatory phenotypes following acute intoxication with the OP, diisopropylfluorophosphate (DFP). Adult male and female Sprague–Dawley rats were administered a single dose of DFP (4 mg/kg, sc) followed by standard medical countermeasures. Within minutes, animals developed benzodiazepine-resistant SE as determined by monitoring seizures using a modified Racine scale. At 1, 3, 7, 14, and 28 d post-exposure (DPE), neuroinflammation was assessed using translocator protein (TSPO) positron emission tomography (PET) and magnetic resonance imaging (MRI). In both sexes, we observed consistently elevated radiotracer uptake across all examined brain regions and time points. A separate group of animals was euthanized at these same time points to collect tissues for immunohistochemical analyses. Colocalization of IBA-1, a marker for microglia, with iNOS or Arg1 was used to identify pro- and anti-inflammatory microglia, respectively; colocalization of GFAP, a marker for astrocytes, with C3 or S100A10, pro- and anti-inflammatory astrocytes, respectively. We observed shifts in the inflammatory profiles of microglia and astrocyte populations during the first month post-intoxication, largely in hyperintense inflammatory lesions in the piriform cortex and amygdala regions. In these areas, iNOS^+^ proinflammatory microglial cell density peaked at 3 and 7 DPE, while anti-inflammatory Arg1^+^ microglia cell density peaked at 14 DPE. Pro- and anti-inflammatory astrocytes emerged within 7 DPE, and roughly equal ratios of C3^+^ pro-inflammatory and S100A10^+^ anti-inflammatory astrocytes persisted at 28 DPE. In summary, microglia and astrocytes adopted mixed inflammatory phenotypes post-OP intoxication, which evolved over one month post exposure. These activated cell populations were most prominent in the piriform and amygdala areas and were more abundant in males compared to females. The temporal relationship between microglial and astrocytic responses suggests that initial microglial activity may influence delayed, persistent astrocytic responses. Further, our findings identify putative windows for inhibition of OP-induced neuroinflammatory responses in both sexes to evaluate the therapeutic benefit of anti-inflammation in this context.

**Supplementary Information:**

The online version contains supplementary material available at 10.1186/s12974-024-03272-8.

## Introduction

Organophosphates (OPs) are a class of compounds used as both insecticides and nerve agents. While diverse in structure and physicochemical properties, most neurotoxic OPs inhibit the enzyme acetylcholinesterase, a critical negative regulator of cholinergic neurotransmission throughout the body [1]. Inhibition of acetylcholinesterase causes excessive cholinergic signaling and can trigger a toxidrome referred to as a cholinergic crisis, characterized by parasympathomimetic symptoms and life-threatening seizures [2]. Current medical countermeasures for cholinergic crisis can increase survival following acute OP poisoning but provide minimal to no protection against chronic adverse neurologic consequences [3, 4]. Both preclinical and clinical studies demonstrate electroencephalographic abnormalities, persistent neuropathologic changes, including neuroinflammation, neurodegeneration, and mineralization, and/or cognitive impairment following acute OP intoxication [4–9].

While numerous pathologic mechanisms are proposed to mediate the adverse neurological outcomes associated with acute OP intoxication [10], increasing evidence points to neuroinflammation as a conserved response and potential driver of long-term neurological sequelae [10, 11]. Preclinical models of acute OP intoxication demonstrate a persistent neuroinflammatory response detectable through histological, biochemical, and in vivo imaging techniques. This neuroinflammatory response is characterized by gliosis and a proinflammatory milieu [12–15] that evolves over a course of hours to months post-exposure [8, 15–18]. Notably, OP-induced neuroinflammation precedes the onset of and persists throughout the development of chronic neurological outcomes, including spontaneous recurrent seizures (SRS) [8] and delayed neurodegeneration [7], and is resistant to standard medical countermeasures [12, 13, 19–26].

The neurotoxic and excitatory nature of inflammatory mediators found in non-OP models of *status epilepticus* (SE) [27] suggest that neuroinflammation may contribute to the neuropathology, SRS and cognitive impairment associated with acute OP intoxication. Indeed, inhibiting proinflammatory pathways reduces neuroinflammation and neurodegeneration following acute OP intoxication [28–31]. Likewise, inhibition of prooxidant inducible nitric oxide synthase (iNOS) reduces neuroinflammation and neurodegeneration and attenuates SRS in the first weeks following SE triggered by the OP diisopropylfluorophosphate (DFP) [16]. While these treatments can improve neuropathological outcomes, they do not reliably or robustly improve chronic seizure and cognitive-behavioral outcomes evaluated months post-exposure.

Context influences the impact of neuroinflammation on central nervous system (CNS) recovery following insult, and accumulating evidence indicates that some inflammatory processes may be beneficial while others are harmful [32, 33]. It is now appreciated that the relative neuroprotective *vs*. neurotoxic role of inflammatory mediators may change over time following the initial insult [11, 34, 35]. For example, depletion of microglia, the brain’s resident immune cells, prior to injury exacerbates damage in several models of CNS insult, including pilocarpine-induced SE [36–38], while microglial depletion later in the inflammatory response improves recovery [37–39]. Similarly, anti-inflammatory pretreatment or administration during or within one hour of SE frequently worsens seizure activity and/or subsequent neuropathology [40–44]. However, delayed anti-inflammatory treatment beginning 6–8 h following control of SE produces more consistent improvements in SE-associated outcomes [43–46].

Microglia and astrocytes are primary cellular mediators of the neuroinflammatory response and both cell types are implicated in neuroprotective and pathological processes [37, 38, 47–50]. Following CNS injury, these cells are activated in a manner dependent on the nature and severity of the insult [51, 52]. Microglia and astrocytes adopt phenotypic states on a pro- to anti-inflammatory continuum in a process known as polarization. Modulation of glia toward a more anti-inflammatory state improves outcomes in SE models [53] and represents a promising therapeutic direction in the context of epilepsy [54].

There is some evidence regarding dynamic shifts in pro- and anti-inflammatory glial phenotypes over time post-SE [55], suggesting an evolving inflammatory landscape following seizurogenic insult. Such data imply that the efficacy of therapeutic interventions may be critically tied to both the timing of administration relative to insult and the pro- or anti-inflammatory state of glial cells at that time. This framework advocates for high temporal resolution of glial inflammatory phenotypes post-insult to guide timing of therapeutic intervention. Shifts in pro- and anti-inflammatory transcripts in microglia and astrocytes over the initial hours to days after OP poisoning have been previously reported in a mouse model [56]; however, whether these transcriptional changes are predictive of cellular changes was not examined, nor has the inflammatory phenotype of microglia and astrocytes been comprehensively characterized in the rat model of acute OP intoxication, which more closely mimics the human neuroinflammatory response [57]. Our study addresses these data gaps, providing a natural history of the spatiotemporal progression of phenotypic changes in both microglia and astrocytes in a rat model of acute OP intoxication with DFP.

## Materials and methods

DFP was purchased from Sigma (St. Louis, MO, USA) and stored at -80 °C. DFP purity of ≥ 90% was confirmed by nuclear magnetic resonance, as previously described [58]. Atropine sulfate (AS) and 2-pralidoxime (2-PAM) were purchased from Sigma and stored at room temperature. Manufacturer certificates of analysis indicated the purity of AS (lot #BCBM6966V) to be > 97% and 2-PAM (lot #MKCG3184) to be > 99%. Pharmaceutical grade midazolam (MDZ, purity ≥ 97%) was purchased from West-Ward Pharmaceuticals (Eatontown, NJ, USA).

All experiments involving animals were performed in accordance with the National Institutes of Health Guide for the Care and Use of Laboratory Animals following protocols approved by the University of California, Davis (UC Davis), Institutional Animal Care and Use Committee. Adult male and female Sprague–Dawley rats (200–225 g) (Charles River Laboratories, Hollister, CA) were individually housed in plastic shoebox cages with corncob bedding under controlled environmental conditions (22 ± 2 °C, 40–50% humidity, 12 h light–dark schedule). Food (2018 Teklad Global 18% Protein Rodent Diet) and water were provided ad libitum. Animals were allowed to acclimate for at least 7 d prior to experimentation.

**Fig. 1:**
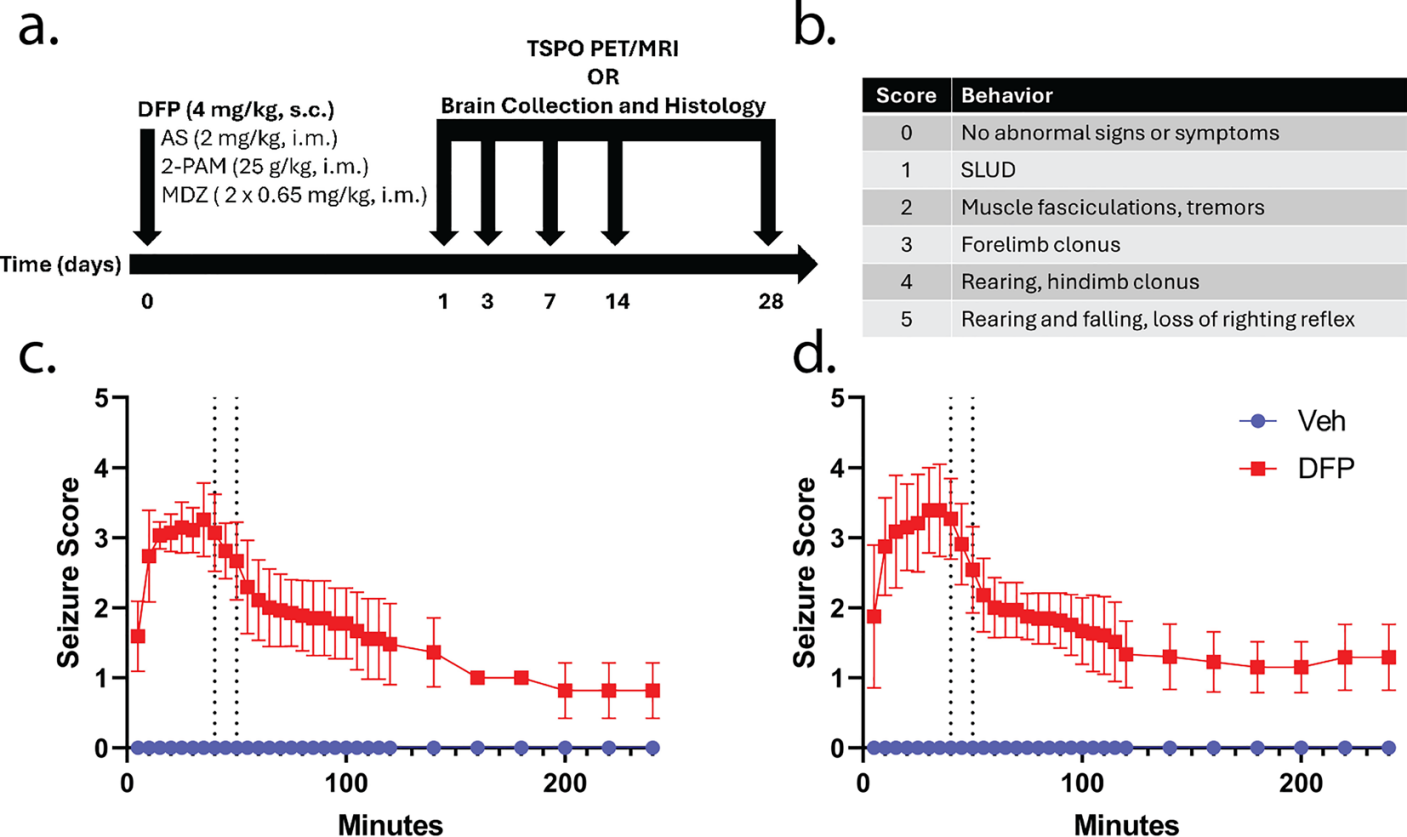
**a** Adult male and female Sprague–Dawley rats were administered DFP followed one min later by atropine sulfate (AS) and 2-pralidoxime (2-PAM). Control animals were administered vehicle (Veh) in place of DFP. At 40 and 50 min post-DFP intoxication, animals received midazolam (MDZ). Behavioral seizures were monitored over the first 4 h post-intoxication. At 1, 3, 7, 14, and 28 d post exposure (DPE), animals were either subjected to translocator protein (TSPO) positron emission tomography (PET) and magnetic resonance imaging (MRI) or euthanized and brains harvested for histological analyses. **b** Modified Racine scale used to evaluate behavioral seizures. Only those animals with an average seizure score of ≥ 2.5 prior to MDZ intervention were included for the remainder of the study (5/42 males and 5/42 females were excluded). **c**, **d** Behavioral seizure scores in males (**c**) and females (**d**) after administration of DFP or Veh. Vertical dotted lines indicate the timing of MDZ treatment. Data are presented as mean ± SD (n = 20–21 Veh and 37 DFP animals per sex)

The DFP intoxication paradigm used in these studies is shown in Fig. 1a. Briefly, on the day of experimentation, a random number generator was used to assign individual animals to experimental groups. DFP was diluted in ice-cold phosphate-buffered saline (PBS) immediately before administration. Animals were injected with DFP (4 mg/kg, s.c.) or an equal volume (300 µl) of vehicle (Veh), which was PBS. One minute later, both DFP and Veh animals were provided a combined injection of AS (2 mg/kg, i.m.) and 2-PAM (25 mg/kg, i.m.) in sterile saline (0.9% NaCl) to reduce mortality from peripheral parasympathetic symptoms in the DFP-intoxicated animals [59]. At 40 and 50 min post-DFP or Veh, animals were provided MDZ (0.65 mg/kg, i.m.). This MDZ dosing paradigm reflects the current standard of care for humans experiencing OP-induced seizures (Chemical Hazards Emergency Medical Management, https://chemm.hhs.gov/na_hospital_mmg.htm#top). Seizure behavior was monitored for 4 h following DFP or Veh administration using a modified Racine scale (Fig. 1b-d). Of the animals intoxicated with DFP, only those with behavior consistent with SE, an average Racine score ≥ 2.5 over 40 min post-DFP, were included in the study [60]. Five males and five females were excluded due to insufficient behavioral seizure scores. After the 4 h monitoring period, animals were provided moistened chow and administered supplemental fluids (5% dextrose in Ringer’s lactate, 10 ml, s.c. (Baxter Healthcare Corporation, Deerfield, IL, USA, up to twice daily until they began to gain weight. A total of 14 males and 7 females died or were euthanized for humane reasons following acute DFP intoxication. Final animal numbers for in vivo imaging and histological analysis were as follows: males: 37 DFP, 20 Veh, females: 37 DFP, 21 Veh.

### In vivo imaging and analysis

Longitudinal positron emission tomography (PET) experiments using the [^18^F]DPA-714 radiotracer, a marker for translocator protein (TSPO) expression, and magnetic resonance imaging (MRI) for anatomic reference were performed at the UC Davis Center for Molecular and Genomic Imaging at 3, 7, 14, and 28 d post-DFP or Veh exposure. A separate group of animals was subjected to TSPO PET and MRI at 1 DPE. At the time of imaging, animals were anesthetized with isoflurane (Piramal Healthcare, Bethlehem PA, USA) / O_2_ using 2.0–3.0% v/v to induce anesthesia and 1.0–2.0% v/v to maintain anesthesia. Anesthetized animals were placed in a stereotaxic head holder consisting of ear and bite bars to prevent motion. The animal’s body temperature was maintained at 37 °C using warmed air, and anesthesia was adjusted to maintain a respiration rate of 50–70 breaths per min.

Automated synthesis of [^18^F]DPA-714 was performed according to previously described methods [61]. PET data were acquired as previously described [14] on one of two preclinical PET systems (Inveon Dedicated PET or microPET Focus 120; both Siemens Medical Solutions, Knoxville, TN, USA). Animals were randomly assigned to each scanner such that treatment groups were divided evenly between scanners, and each animal was imaged on the same scanner at all time points. Prior to image acquisition, [^18^F]DPA-714 (~ 37 MBq in 200 μl of saline) was delivered by tail-vein injection. After a 30 min radiotracer uptake period, a 30 min scan was acquired (30–60 min post [^18^F]DPA-714 injection)*.* PET data were reconstructed using two iterations of the 3-D ordered subset expectation maximization (OSEM3D) method followed by 18 iterations of a maximum a posteriori (MAP) algorithm into a single 30 min frame. The reconstruction matrix was 128 × 128 × (95 or 159; scanner dependent) with reconstructed voxel sizes of 0.78 × 0.78 × 0.80 mm^3^.

**Fig. 2:**
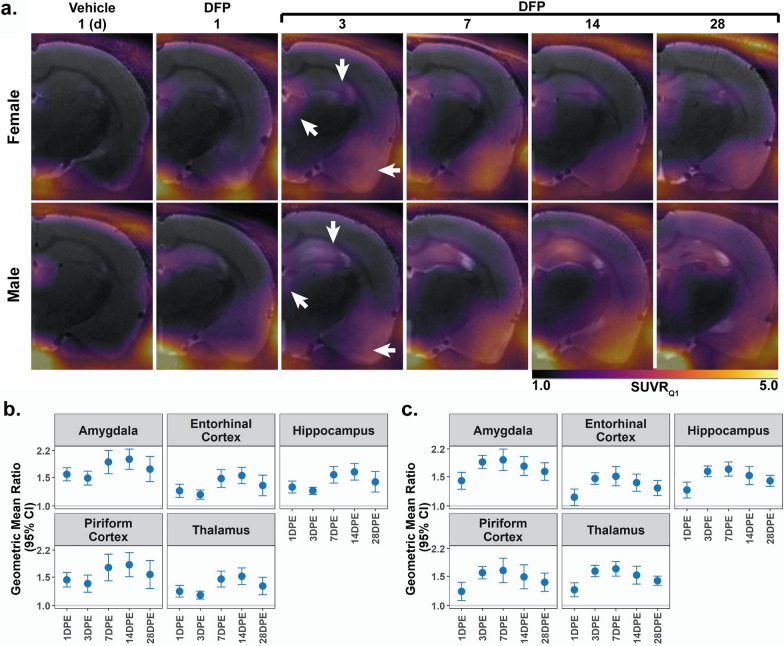
**a** Longitudinal [^18^F]DPA-714 PET SUVR_Q1_ maps. Data are overlaid on corresponding T_2_-weighted images from the same animals. The [^18^F]DPA-714 uptake observed in the Veh animal at 1 DPE (left column, also Supplementary Fig. 1) is largely due to non-specific binding. By comparison, male and female DFP animals (right columns) display significant radiotracer uptake in the amygdala and piriform cortex (left arrow), hippocampus (down arrow), and thalamus (up-left arrow). In all scans there is minor signal penetration from outside the brain along the skull and jaw bones due to [^18^F] binding in bone following defluorination of the radiotracer. **b**, **c** Regional [^18^F]DPA-714 uptake data quantified by standard uptake value (SUV) analysis normalized to the SUV derived from the first quartile of a cerebellar reference region (SUVR_Q1_). Geometric mean ratio (GMR, dot) of the mean SUVR_Q1_ with 95% confidence intervals (CIs, bars) in (**b**) male (n = 3–4 Veh; 7–9 DFP) and (**c)** female (n = 3–5 Veh, 6–9 DFP). CIs that do not include 1 (the gray horizontal line) and are shaded blue indicate a significant difference between SUVR_Q1_ in DFP vs. Veh after FDR correction

PET images were co-registered to sequentially-acquired T_2_ weighted (T_2_w) MRI using PMOD v4.4 (PMOD Technologies, Zurich, Switzerland), and the volumes of interest (VOIs) derived from MR images were transferred to the PET data. For each VOI, the standardized uptake values (SUV) and SUV normalized to the average SUV of the lowest quartile of voxels in the cerebellum (SUV ratio, SUVR_Q1_) were calculated for the 30 min frame data. Normalization to a cerebellar reference region has been validated previously [14, 15]. The average SUV of the lowest quartile of voxels in the cerebellum was utilized to mitigate aberrant signal penetration from [^18^F] uptake in the skull due to tracer defluorination, typical of TSPO radiotracers (Fig. 2a).

MRI scans were performed as previously described [20] using a Bruker Biospec 70/30 (7 T) preclinical MR scanner running Paravision 6.0 (Bruker BioSpin MRI, Ettlingen, Germany), equipped with a 116 mm internal diameter B-GA12S gradient (450 mT/m, 4500 T/m/s), a 72-mm internal diameter linear transmit coil, and a four-channel, rat-brain phased array in cross-coil configuration for signal reception. Multi-slice, T_2_w, fast spin echo (Rapid Acquisition with Repeated Echoes, RARE) axial images were acquired using the following parameters: repetition time (TR) = 4500 ms; echo time (TE) = 8.5 ms; RARE factor = 8; averages = 4; field of view (FOV) = 30 × 30 mm^2^, with an in-plane data matrix of 240 × 240, resulting in a data set resolution of 0.125 × 0.125 mm^2^; 55 slices with a 0.5 mm thickness spanning approximately 9 mm to − 17.0 mm bregma. Dynamic contrast-enhanced MRI data were also acquired; these data are discussed in a separate manuscript. Total imaging time for each animal was approximately 60 min when accounting for animal positioning within the scanner and data acquisition.

Brain region VOIs were delineated utilizing a fully-automated image analysis pipeline and rat brain atlas. First, rat brain scans underwent whole brain delineation and masking (“skull stripping”) using a modified 2D U-Net-based convolutional neural network trained specifically on brain scans of DFP-intoxicated, DFP with multiple treatments, and Veh control rats [62]. Next, isolated brain volumes underwent fully-automated co-registration (utilizing rigid and non-rigid warping constraints) to an in-house rat brain atlas based on the Paxinos and Watson’s *The Rat Brain in Stereotaxic Coordinates* [63] using the Elastix module within 3D Slicer (http://www.slicer.org) [64–66]. Brain region VOIs used for the present study were inspected for artifacts and manually adjusted as needed.

### Tissue collection

On 1, 3, 7, 14, and 28 d post-exposure (DPE), animals not subjected to in vivo imaging were deeply anesthetized with 5% isoflurane in medical grade oxygen at a flow rate of 0.5 l/min and once deeply anesthetized, transcardially perfused with PBS at a flow rate of 15 ml/min using a Masterflex peristaltic pump (Cole Parmer, Vernon Hills, IL, USA). Brains were sectioned into 2 mm thick coronal sections using a stainless-steel small rat brain matrix (Zivic Instruments, Pittsburgh, PA, USA). Sections were post fixed in 4% (w/v) paraformaldehyde (Sigma) in 0.1 M phosphate buffer for 24 h and then cryoprotected in 30% (w/v) sucrose in PBS for an additional 72 h. Fixed sections were embedded in Optimal Cutting Temperature (OCT) medium (Thermo Fisher Scientific, Waltham, MA, USA) and stored at -80 °C. Tissue blocks were cryosectioned at a thickness of 10 µm from bregma − 3.3 to − 4.2 onto Superfrost Plus Slides (Thermo Fisher Scientific) and stored at − 20 °C until immunostained.

### Immunohistochemistry

Cryosections were equilibrated to room temperature and washed in 0.03% (v/v) Triton X-100 (Thermo Fisher Scientific) in PBS (T-PBS). Slides for immunolabeling protocols that required antigen retrieval were placed in 10 mM sodium citrate buffer at pH 6.0 and heated for 30 min at 95 °C in a Decloaking Chamber NxGen (Model Dc2012, Biocare Medical, Pacheco, CA, USA). After antigen retrieval, tissues were washed 3X for 5 min with T-PBS and then blocked for 1 h in PBS containing an immunologically compatible blocking buffer: either 10% (v/v) normal goat serum (Vector Laboratories, Burlingame, CA, USA; RRID: AB_2336615) or 5% (v/v) normal donkey serum (Abcam, Waltham, MA, USA), 1% (w/v) bovine serum albumin (Sigma), and 0.03% or 0.1% (v/v) Triton X-100 (Thermo Fisher Scientific). Slides that did not require antigen retrieval were washed once for 5 min with T-PBS and incubated with blocking buffer as above. Depending on immunolabeling protocol, sections were incubated with primary antibody for 1 h at room temperature (mouse anti-C3/guinea pig anti-GFAP) or overnight at 4 °C (all other primary antibody combinations), washed three times for 5 min with 0.03% T-PBS, incubated for 1 h with secondary antibody, washed three times for 5 min with PBS, and mounted in ProLong Gold Antifade with DAPI (Invitrogen, Waltham, MA, USA). For each immunolabeling protocol, 2–4 technical replicates were performed for each animal. Primary antibodies included: rabbit anti-IBA1 (1:1000, RRID: AB_839504), goat anti-IBA1 (1:500, RRID:AB_521594), rabbit anti-iNOS (1:100, RRID:AB_301857), goat anti-Arg1 (1:100, RRID:AB_892299), guinea pig anti-GFAP (1:500, RRID:AB_10641162), mouse anti-C3 (1:100, RRID:AB_627277), and mouse anti-S100A10 (1:200, RRID:AB_2717244). Secondary antibodies included: goat anti-mouse (1:500, RRID:AB_141611), goat anti-guinea pig (1:500, RRID:AB_2534117), goat anti-mouse IgG2a (1:500, RRID:AB_2535810), donkey anti-goat IgG (1:500, RRID:AB_2534102), and donkey anti-rabbit IgG (1:500, RRID: AB_2534017).

Fluorescent images of the somatosensory cortex, CA1 hippocampus, CA3 hippocampus, dorsolateral thalamus, amygdala, and piriform cortex were acquired at 20 × magnification using the high-content ImageXpress XL imaging system (Molecular Devices, Sunnyvale, CA, USA) and analyzed using custom scripts in ImageJ [67]. These brain regions experience the most severe neuronal necrosis, microgliosis, and astrogliosis following acute DFP intoxication [15]. Colocalization of IBA-1, a marker for microglia, with iNOS or Arg1 was used to identify pro- and anti-inflammatory microglia, respectively [68–70]. Colocalization of GFAP, a marker for astrocytes, with C3 or S100A10 identified pro- and anti-inflammatory astrocytes, respectively [71–74]. Microglial and astrocytic nuclei count were obtained by identifying DAPI-positive objects that colocalized ≥ 70% with IBA-1- or ≥ 40% GFAP-immunopositive area, respectively. Nuclei density was determined by normalization to the area of the region of interest. Phenotypic markers were identified through application of the Phansalkar Auto Local Threshold calculated using a radius of 5% of the image width. For each phenotypic marker, immunopositive area was colocalized with microglial or astrocytic nuclei to produce a count of each glial phenotype. Density of each cell type was determined by normalization to the region area.

### Statistical analysis

For in vivo imaging, the primary outcomes were region-specific SUV and SUVR_Q1_. For histology, the primary outcomes were IBA1 or GFAP nuclei density and polarized IBA1 or GFAP nuclei density. Mixed effects models, including animal-specific random effects, were fit separately by sex to assess differences between DFP and Veh. Primary factors of interest included exposure (DFP, Veh), brain region, and time point (1, 3, 7, 14, 28 DPE). Interactions between the factors (exposure, region, time point) were considered, and the best model was chosen using Akaike Information Criterion. The outcomes were transformed using the natural logarithm to better meet the assumptions of the model; the mean intensity of C3 labeling on GFAP nuclei was shifted by 10 prior to the natural logarithm transformation. Contrasts for differences between exposure groups either overall or by region or time point were constructed and tested using a Wald test. The Benjamini–Hochberg false discovery rate (FDR) was used to account for multiple comparisons. Results are presented as geometric mean ratios (GMR) between exposure groups. Point estimates of the ratios and 95% confidence intervals are presented in the figures. When the confidence interval for the GMR includes 1, there is no statistical evidence of a difference between groups. All analyses were performed using SAS software, version 9.4, and alpha was set at 0.05. Comparisons remained statistically significant after the FDR correction unless otherwise stated.

## Results

### TSPO response to neuroinflammation detected by PET

We assessed progression of TSPO expression in male and female rats intoxicated with DFP by subjecting a subset of animals to [^18^F]DPA-714 PET at various timepoints following DFP exposure. At all timepoints post-DFP intoxication, inspection of PET data identified elevated radiotracer uptake in both males and females across numerous cortical and subcortical areas (Fig. 2a). Comparative PET data for Veh are provided as Supplementary Fig. 1. In both sexes, acute DFP intoxication produced consistent increases in SUVR_Q1_ in all brain regions and at all time points relative to Veh (males: p < 0.001; females: p < 0.04) (Fig. 2b, c).

### Microglia cell density

**Fig. 3:**
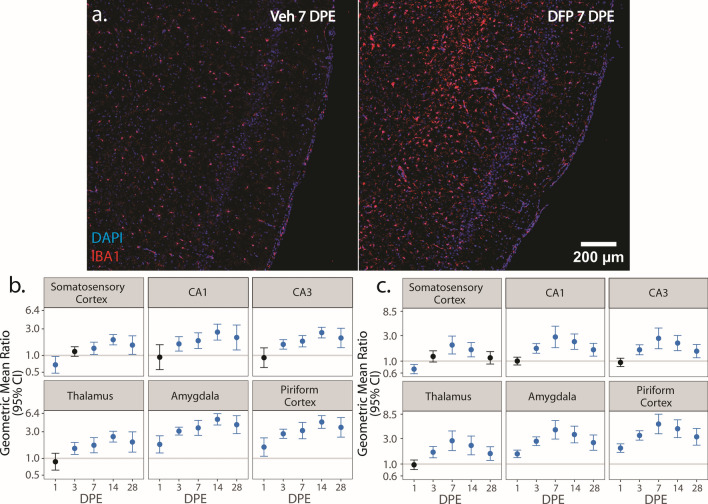
**a** Representative photomicrographs of piriform cortex-amygdala immunostained for IBA1 (red) to identify microglia and counterstained with DAPI (blue) to identify cell nuclei. **b**, **c** Geometric mean ratio (GMR) (dot) of the mean density of IBA1^+^ cells in various brain regions of animals intoxicated with DFP relative to Veh at 1, 3, 7, 14, and 28 DPE with 95% confidence intervals (bars) in males (**b**) and females (**c**). The y-axis is shown as a log-scale. Confidence intervals that do not include 1 (the gray horizontal line) and are shaded blue indicate a significant difference in the density of IBA1^+^ nuclei between DFP and Veh after FDR correction

To identify microglial cell populations and evaluate phenotypic polarization of microglia, we co-labeled brain sections for IBA1 with either iNOS or Arg1 to identify pro-inflammatory and anti-inflammatory phenotypes [68, 69]. Both protocols yielded comparable data regarding the density of IBA1-immunopositive nuclei. For clarity, the density of IBA1 nuclei is reported in the main text only from sections co-labeled with iNOS (Fig. 3; Supp. Figure 2a, b). Comparable data from sections co-labeled for IBA1 and Arg1 are provided in the supplemental material (Supp. Figure 3).

DFP animals of both sexes displayed differences in the density of IBA1 nuclei compared to Veh controls that varied by brain region and DPE (Fig. 3). At 1 DPE, DFP animals of both sexes demonstrated increased density of IBA1 nuclei in the amygdala and piriform cortex, but decreased density in the somatosensory cortex (Fig. 3b, c; Supp. Figure 2a, b). By 3 DPE, DFP animals displayed increased density of IBA1 nuclei in all brain regions examined (male: p < 0.002; female: p < 0.001) except for the somatosensory cortex (p = 0.1) (Fig. 3b, c; Supp. Figure 2a, b). By 7 DPE, the density of IBA1 nuclei was higher in DFP than Veh in all assessed brain regions (male: p < 0.025; female: p < 0.001) and remained elevated at 14 (male: p < 0.001; female: p < 0.002) and 28 (male: p < 0.03; female: p < 0.005) DPE in all brain regions with the exception of the somatosensory cortex in females at 28 DPE (p = 0.3) (Fig. 3b, c; Supp. Figure 2a, b).

### Microglial inflammatory phenotypes

**Fig. 4:**
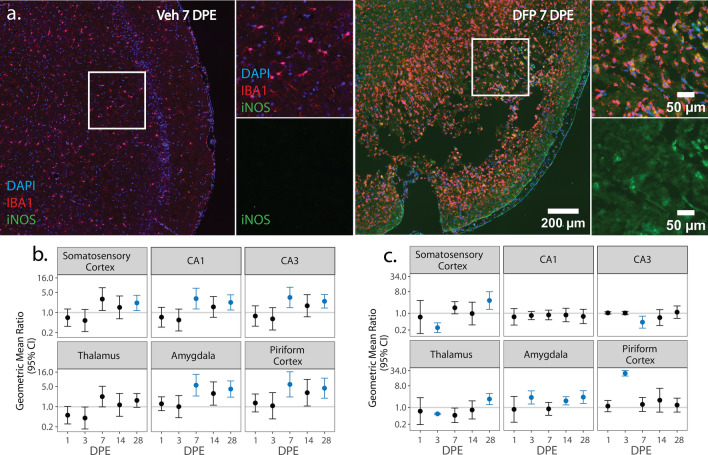
**a** Representative photomicrographs of piriform cortex-amygdala immunostained for IBA1 (red) to identify microglia and iNOS (green) to identify pro-inflammatory cells, then counterstained with DAPI (blue) to identify cell nuclei. Solid boxes identify the field in the lower magnification image that is shown at higher magnification. **b**, **c** (GMR) (dot) of the mean density of iNOS^+^ IBA1^+^ cells in various brain regions of animals intoxicated with DFP relative to Veh at 1, 3, 7, 14, and 28 DPE with 95% CI (bars) in males (**b**) and females (**c**). The y-axis is shown as a log-scale. CI that does not include 1 (the gray horizontal line) and is shaded blue indicates a significant difference in the density of iNOS^+^ IBA1^+^ nuclei between DFP and Veh after FDR correction

**Fig. 5:**
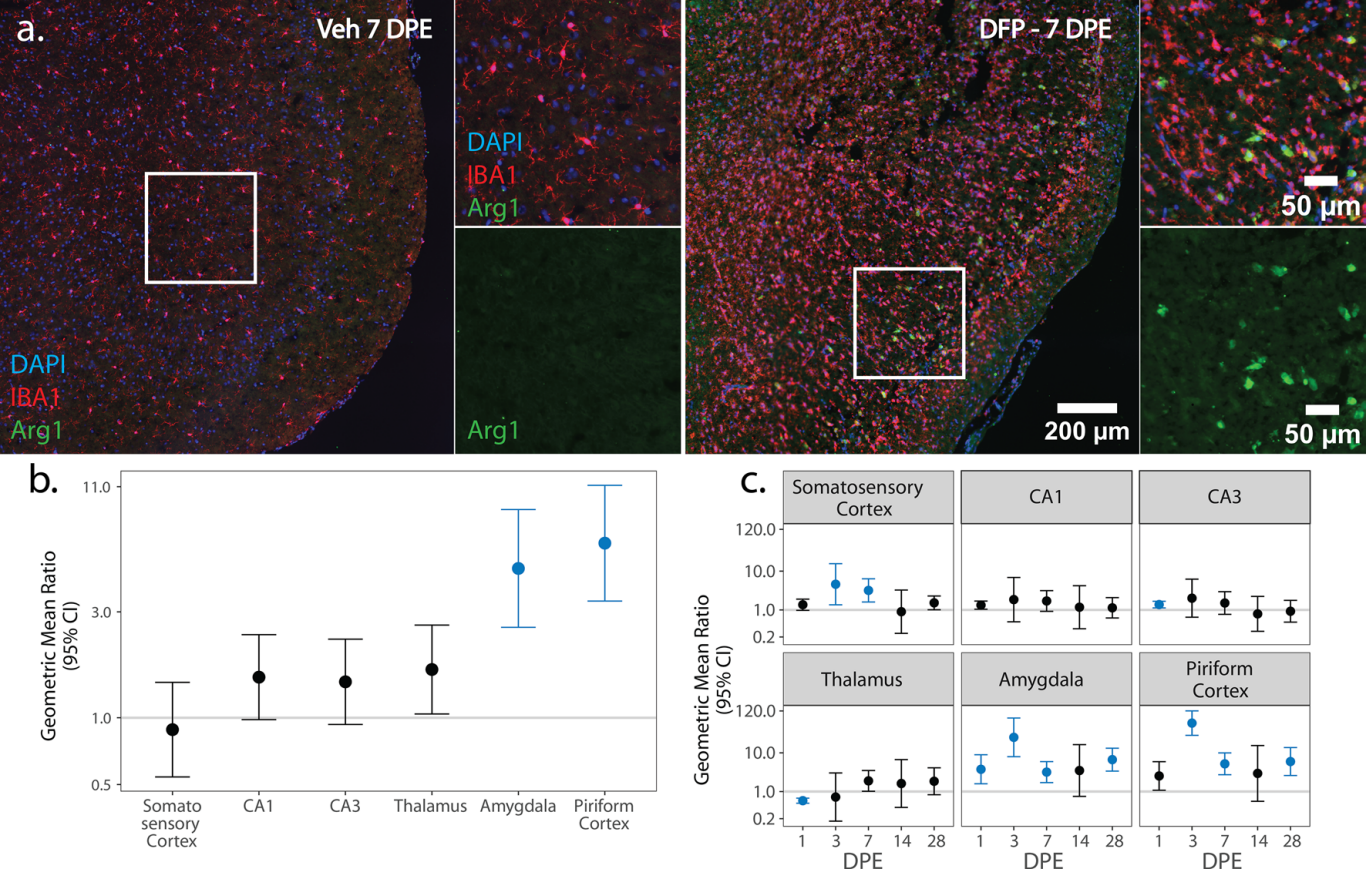
**a** Representative photomicrographs of piriform cortex-amygdala immunostained for IBA1 (red) to identify microglia and Arg1 (green) to identify anti-inflammatory cells, then counter-stained with DAPI (blue) to identify cell nuclei as observed at lower and higher magnification. Solid boxes identify the field in the lower magnification image that is shown at higher magnification. **b**, **c** GMR (dot) of the mean density of Arg1^+^ IBA1^+^ nuclei in in various brain regions of animals intoxicated with DFP relative to Veh at 1, 3, 7, 14, and 28 DPE with 95% CI (bars) in males (**b**) and females (**c**). **b** In males, differences in Arg1^+^ IBA1^+^ nuclei density did not vary with DPE so overall difference by brain region is displayed. The y-axis is shown as a log-scale. A CI that does not include 1 (the gray horizontal line) and is shaded blue indicates a significant difference in the density of Arg1^+^ IBA1^+^ nuclei between DFP and Veh after FDR correction

We next assessed the spatiotemporal distribution of pro- and anti-inflammatory microglia as identified via colocalization of IBA1 with iNOS (Fig. 4) and Arg1 (Fig. 5), respectively.

iNOS is a pro-oxidant enzyme associated with pro-inflammatory myeloid cell polarization [68]. iNOS-immunopositive IBA1 cells were not detected in the Veh or DFP brain in significant numbers until 3 DPE, at which time relative to Veh controls, the density of iNOS IBA1 nuclei in DFP females was increased in the amygdala (p = 0.003) and piriform cortex (p < 0.001) but decreased in the somatosensory cortex (p < 0.001) and thalamus (p < 0.001) (Fig. 4c; Supp. Figure 2d). In contrast, at 3 DPE, DFP males did not exhibit significant changes in pro-inflammatory microglia density in any of the assessed brain regions. By 7 DPE, the density of iNOS^+^ IBA1 nuclei was increased in DFP males in the CA1 (p = 0.009), CA3 (p = 0.004), amygdala (p < 0.001), and piriform cortex (p < 0.001) (Fig. 4b; Supp. Figure 2c). In contrast, in DFP females, the initial increase in iNOS^+^ IBA1 nuclei density observed at 3 DPE was not significantly different from Veh at 7 DPE; iNOS^+^ IBA1 nuclei density was lower in DFP than Veh in CA3 (Fig. 4c; Supp. Figure 2d). A second wave of pro-inflammatory microglial polarization was observed at 14 DPE in DFP females, which demonstrated increased density of iNOS^+^ IBA1 nuclei in the amygdala (p < 0.002) (Fig. 4c; Supp. Figure 2d). At 28 DPE, DFP brains in males displayed increased density of iNOS^+^ IBA1 nuclei in all brain regions (p < 0.015) except the thalamus (p = 0.07). At 28 DPE, female DFP brains had higher iNOS^+^ IBA1 nuclei density than VEH in the somatosensory cortex (p = 0.005) and thalamus (p = 0.003) (Fig. 4b, c; Supp. Figure 2c, d).

Arg1 is an anti-inflammatory enzyme involved in matrix deposition and wound healing [69]. At all DPE, anti-inflammatory microgliosis was evident in the DFP males with DFP animals exhibiting consistently increased density of Arg1^+^ IBA1 nuclei in the amygdala (p < 0.001) and piriform cortex (p < 0.001) relative to Veh (Fig. 5b; Supp. Figure 2e). In contrast, at 1 DPE, DFP females showed increased density of Arg1^+^ IBA1 nuclei in the CA3 and amygdala (p < 0.003), but decreased density in the thalamus (p < 0.001). At 3 DPE, the density of Arg1^+^ IBA1 nuclei was higher in DFP females in the somatosensory cortex (p = 0.01), amygdala (p < 0.001), and piriform cortex (p < 0.001, and these values remained elevated in the amygdala and piriform at 7 DPE (p < 0.015). At 28 DPE, anti-inflammatory microgliosis was observed in females again in the amygdala and piriform cortex (p < 0.001) (Fig. 5c; Supp. Figure 2f).

### Astrocytic cell density

**Fig. 6:**
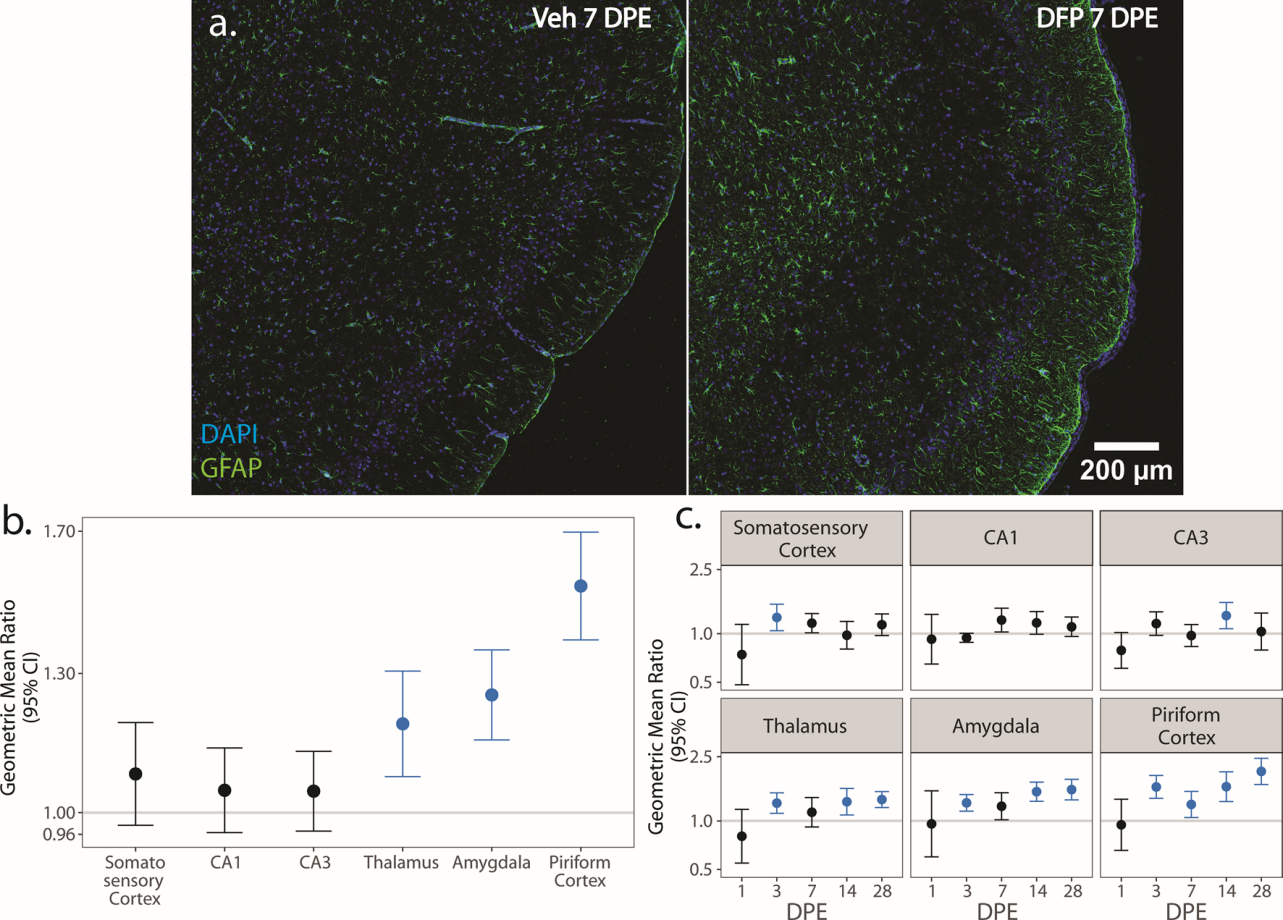
**a** Representative photomicrographs of piriform cortex-amygdala immunostained for GFAP (green) to identify astrocytes and counterstained with DAPI (blue) to identify cell nuclei. **b**, **c** GMR (dot) of the mean density of GFAP^+^ nuclei in in various brain regions of animals intoxicated with DFP relative to Veh at 1, 3, 7, 14 and 28 DPE with 95% CI (bars) in males (**b**) and females (**c**). **b** In males, differences in GFAP^+^ nuclei density did not vary across DPE, so overall difference by brain region is displayed. The y-axis is shown as a log-scale. A CI that does not include 1 (the gray horizontal line) and is shaded blue indicates a significant difference in the density of GFAP^+^ nuclei between DFP and Veh after FDR correction

To identify pro- and anti-inflammatory astrocytic cell populations and evaluate aspects of astrogliosis, we co-labeled brain sections with GFAP and either C3 or S100A10 [71–74], respectively. Both protocols yielded comparable data regarding the density of GFAP immunopositive nuclei. For clarity, the density of GFAP nuclei is reported in the main text only from samples co-labeled with S100A10 (Fig. 6; Supp. Figure 4a, b). Comparable data from sections co-labeled with GFAP and C3 are provided in the supplemental material (Supp. Figure 5).

The density of GFAP nuclei differed between DFP and Veh in males by brain region (Fig. 6b), with increases in the thalamus (p < 0.001), amygdala (p < 0.001), and piriform cortex (p < 0.001) (Fig. 6b; Supp. Figure 4a). In females, the impact of DFP on the density of GFAP nuclei relative to Veh varied according to brain region and DPE (Fig. 6c). At 1 DPE, there were no significant differences between DFP and Veh in any brain region (p > 0.07). By 3 DPE, the density of GFAP nuclei was increased in DFP relative to Veh in all brain regions (p < 0.025) except for the CA1 (p = 0.07) and CA3 (p = 0.09). At 7 DPE, GFAP nuclei density was higher in DFP females than Veh females in the piriform cortex (p = 0.01). Astrogliosis expanded at 14 DPE, with increased GFAP nuclei density in DFP relative to Veh in the CA3 (p = 0.007), thalamus (p = 0.005), amygdala (p < 0.001), piriform cortex (p < 0.001). At 28 DPE, the density of GFAP nuclei remained higher in DFP females relative to Veh females in the thalamus (p < 0.001), amygdala (p < 0.001), and piriform cortex (p < 0.001) (Fig. 6c; Supp. Figure 4b).

### Astrocyte inflammatory phenotypes

**Fig. 7:**
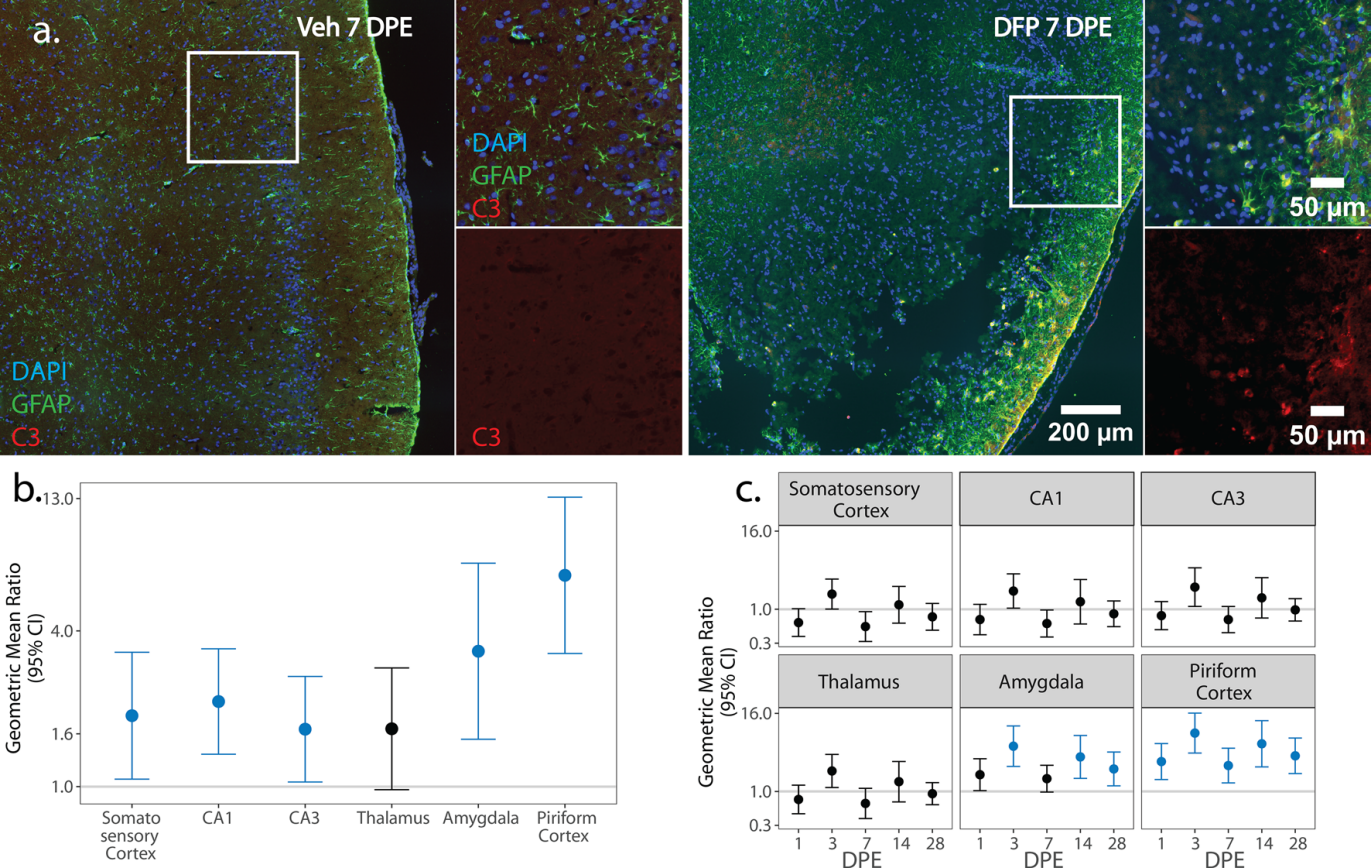
**a** Representative photomicrographs of piriform cortex-amygdala immunostained for GFAP (green) to identify astrocytes, C3 (red) to identify pro-inflammatory cells, and counterstained with DAPI (blue) to identify cell nuclei shown at lower and higher magnifications. Solid boxes identify the field in the lower magnification image that is shown at higher magnification. **b**, **c** Geometric mean ratio (GMR) (dot) of the mean C3^+^ GFAP^+^ nuclei density in various brain regions of animals intoxicated with DFP relative to Veh at 1, 3, 7, 14, and 28 DPE with 95% confidence intervals (bars) in males (**b**) and females (**c**). **b** In males, differences in C3^+^ GFAP^+^ nuclei density did not vary DPE so overall difference by brain region is displayed. The y-axis is shown as a log-scale. Confidence intervals that do not include 1 (the gray horizontal line) and are shaded blue indicate a significant difference in the density of C3^+^ GFAP^+^ nuclei between DFP and Veh after FDR correction

**Fig. 8:**
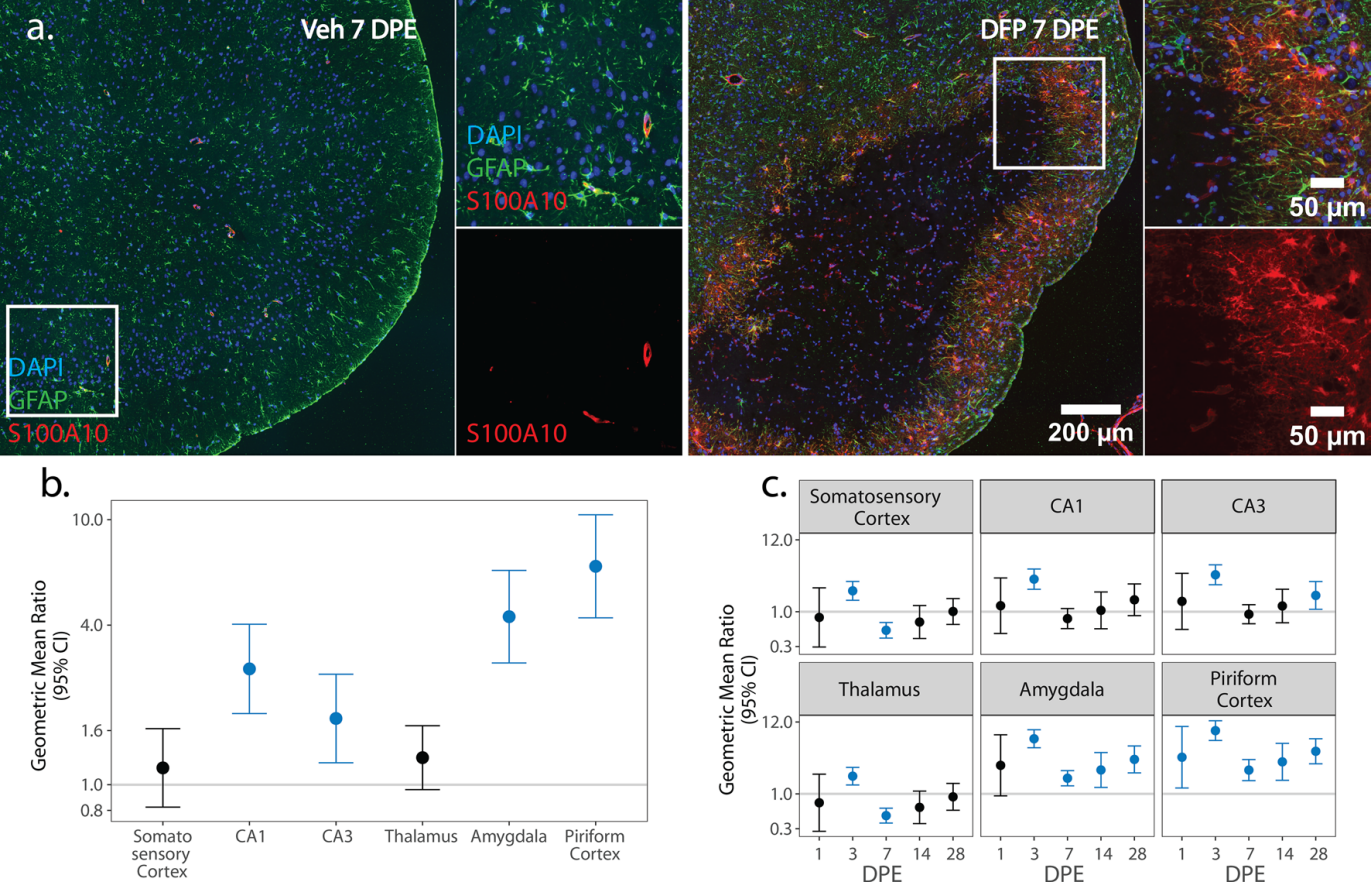
**a** Representative photomicrographs of piriform cortex-amygdala immunostained for GFAP (green) to identify astrocytes and S100A10 (red) to identify anti-inflammatory cells, then counterstained with DAPI (blue) to identify cell nuclei. Solid boxes identify the field in the lower magnification image that is shown at higher magnification. **b**, **c** GMR (dot) of the mean density of S100A10^+^ GFAP^+^ nuclei in various brain regions of animals intoxicated with DFP relative to Veh at 1, 3, 7, 14, and 28 DPE with 95% CI (bars) in males (**b**) and females (**c**). **b** In males, differences in S100A10^+^ GFAP^+^ nuclei density did not vary across DPE, so overall difference by brain region is displayed. The y-axis is shown as a log-scale. A CI that does not include 1 (the gray horizontal line) and is shaded blue indicates a significant difference in the density of S100A10^+^ GFAP^+^ nuclei between DFP and Veh after FDR correction

We next assessed the spatiotemporal distribution of pro- and anti-inflammatory astrocytes via colocalization of GFAP with C3 (Fig. 7) and S100A10 (Fig. 8), respectively. C3 upregulation in astrocytes is downstream of NF-κB signaling and indicative of a neurotoxic and pro-inflammatory phenotype [72, 74]. Across time point and sex, hypertrophic C3^+^ astrocytes concentrated at the boundary of GFAP-depleted areas in the piriform cortex and amygdala (Fig. 7a). In males, the density of pro-inflammatory C3^+^ GFAP nuclei was consistently higher in DFP than Veh in all brain regions (p < 0.035), except for the thalamus (p = 0.06) (Fig. 7b; Supp. Figure 4c). In females, however, differences in the density of C3^+^ GFAP nuclei between DFP and Veh varied by brain region and DPE (Fig. 7c). In DFP females, increased pro-inflammatory astrogliosis was observed as early as 1 DPE in the piriform cortex (p = 0.001). This increased density of C3^+^ GFAP nuclei persisted in the piriform cortex at all remaining time points (3 DPE: p < 0.001; 7 DPE: p = 0.003; 14 DPE: p < 0.001; 28 DPE: p < 0.001) and expanded to the amygdala at 3 (p < 0.001), 14 (p = 0.002), and 28 (p = 0.009) DPE (Fig. 7c; Supp. Figure 4d).

S100A10 is a calcium binding protein associated with an anti-inflammatory astrocytic phenotype [72, 74]. Similar to C3, S100A10^+^ astrocytes were most abundant at the boundary of GFAP-depleted areas in the piriform cortex and amygdala in both sexes (Fig. 8a). In males, the difference in the density of S100A10^+^ GFAP nuclei between DFP and Veh varied by brain region, but not DPE (Fig. 8b). Across all time points, the density of anti-inflammatory astrocytes was increased in DFP males, as determined by higher S100A10^+^ GFAP nuclei density in DFP than Veh in the CA1 (p < 0.001), CA3 (p = 0.003), amygdala (p < 0.001), and piriform cortex (p < 0.001) (Fig. 8b; Supp. Figure 4e).

In contrast, in females, the difference in the density of S100A10^+^ GFAP nuclei between DFP and Veh in females varied by both brain region and DPE (Fig. 8c). Anti-inflammatory astrogliosis was observed in DFP females at 1 DPE, with increased S100A10^+^ GFAP nuclei density in the piriform cortex (p = 0.02). The distribution of S100A10^+^ astrocytes in the DFP brain expanded by 3 DPE, as evident by increased density of S100A10^+^ GFAP nuclei in all brain regions (p < 0.001). At 7 DPE, the density of S100A10^+^ GFAP nuclei was suppressed in the somatosensory cortex (p < 0.001) and thalamus (p < 0.001). S100A10^+^ GFAP nuclei density remained elevated in the amygdala and piriform cortex at all remaining time points (amygdala: 7 DPE: p < 0.001; 14 DPE: p = 0.007; 28 DPE: p < 0.001; piriform cortex: 7 DPE: p < 0.001; 14 DPE: p < 0.001; 28 DPE: p < 0.001). The CA3 also displayed elevated S100A10^+^ GFAP nuclei density at 28 DPE (p = 0.02) (Fig. 8c; Supp. Figure 4f).

## Discussion

Previous studies have documented profound microgliosis and astrogliosis that persists for months following acute OP intoxication [8, 12–18]. These studies have not, however, characterized the presence and number of pro- vs. anti-inflammatory glia during the days to weeks post-intoxication. Immunomodulation is widely posited as a therapeutic strategy for mitigating the adverse neurological consequences of acute OP intoxication, but results of studies that have tested such approaches are equivocal [11]. Interpretation of these discrepant findings is confounded by emerging experimental evidence of an evolving inflammatory landscape with shifts in the inflammatory phenotype of glia over hours to days post-intoxication in mice [56].

A better understanding of the spatiotemporal profile of pro- and anti-inflammatory glial phenotypes in the context of co-occurring neuropathologic processes may better inform therapeutic windows for maximally effective anti-inflammatory interventions. To this end, we provide novel data characterizing the natural history of microglial and astrocytic inflammatory phenotypic shifts over the first month following acute OP intoxication in male and female rats.

### Temporal profiles of pro-inflammatory and anti-inflammatory glial phenotypes

**Fig. 9:**
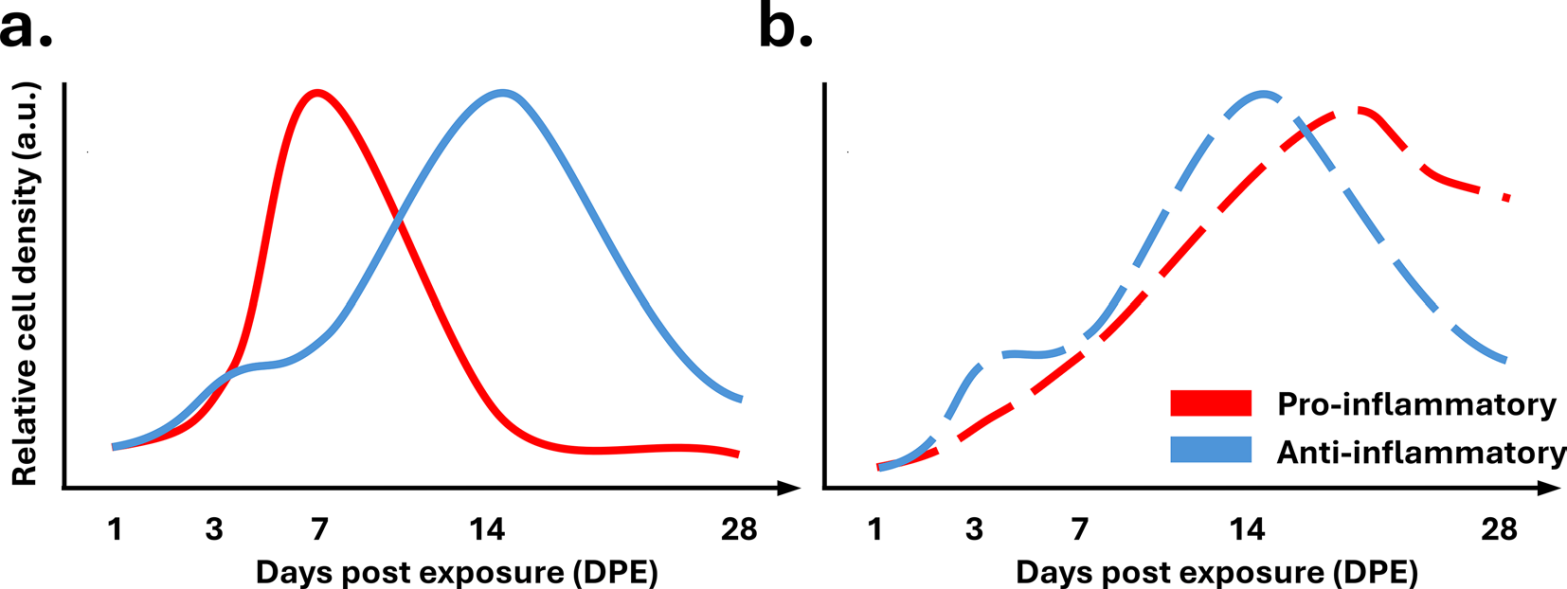
Simplified temporal schematic representing the density of (**a**) microglial and (**b**) astrocytic pro- and anti-inflammatory cells, red and blue, respectively, in the amygdala and piriform cortex of male rats over 28 days following acute DFP intoxication

Over the first month post-DFP intoxication, we observed marked increases in TSPO PET signal in both sexes and across all brain regions evaluated, consistent with previous reports [14, 20]. In contrast, histological assessments revealed brain region-specific shifts in the phenotype of microglia and astrocyte populations in both male and female rats over this same period. In general, astrocytic responses are delayed relative to microglial activation; the density of pro- and anti-inflammatory microglia peaks at 7 and 14 DPE, respectively, while the density of pro- and anti-inflammatory astrocytes is greatest at 14 DPE and remains elevated at 28 DPE (Fig. 9). These observations are in agreement with a sizable literature documenting the spatiotemporal progression of glial responses following acute OP intoxication [11] and suggest that the TSPO response reflects both pro- and anti-inflammatory microglial and astrocytic activation in the DFP rat. While microglia and astrocytes both contribute to the overall TSPO response [75, 76], it is unclear if an upregulation in TSPO reflects a pro- or anti-inflammatory cell phenotype. The origins of the TSPO response in our data are consistent with a previous report of TSPO expression in both pro- and anti-inflammatory cells [77], however, they contrast with other data of TSPO upregulation exclusively in pro-inflammatory cells [78–80]. While such inconsistencies may reflect differences in both model species and neuroinflammatory stimulus, dedicated studies are needed to confirm the origin of the TSPO response following acute OP intoxication in the rat.

We observed mixed inflammatory phenotypes throughout monitoring, conforming with previous findings [56] and as expected from a complex sterile injury; however, population-wide shifts in phenotypic profiles occurred over time post-intoxication. Within the first week post-intoxication, the microglial response included both pro- and anti-inflammatory phenotypes. After the first week post-intoxication, anti-inflammatory microglia dominate. In comparison, astrocytic responses were mixed throughout monitoring, with the greatest density of phenotypically polarized astrocytic cells observed weeks post-DFP intoxication.

Initial microglial activation followed by a delayed astrocytic response conforms with evidence that microglial phenotypes shape subsequent astrocytic activation [74, 81]. Interestingly, we observed persistence of pro-inflammatory astrocytes beyond the initial pro-inflammatory microglia response. Following middle cerebral artery occlusion or systemic lipopolysaccharide administration, polarized astrocytic phenotypes fade within a week of insult [82], but persistence of these inflammatory states in our model implies underlying mechanisms for sustaining astrocytic polarization following acute OP intoxication.

It is possible that a small population of pro-inflammatory microglia is sufficient to stimulate pro-inflammatory astrocytic phenotypes. We observed clustering of pro-inflammatory astrocytes around inflammatory lesions, potentially due to proximity of pro-inflammatory microglia [74]. Despite a microglial population dominated by anti-inflammatory activation, pro-inflammatory microglia are still evident, and adjacency to these cells may be sufficient to sustain pro-inflammatory astrocyte polarization. Our current methods do not allow co-visualization of microglia and astrocytes, limiting evaluation of the spatial relationship between these cell populations. We also do not fully understand the nature of microglial input needed to induce pro-inflammatory astrocyte polarization in the three-dimensional parenchymal space. Further study is needed to answer these unknowns.

Other potential mechanisms underlying the delayed activation of pro-inflammatory astrocytes include stimulation by iNOS-immunonegative microglia or microglia-independent astrocytic polarization. While iNOS upregulation is associated with microglial phenotypes known to induce pro-inflammatory astrocyte polarization [74, 83], a direct connection between microglial iNOS-immunoreactivity and pro-inflammatory astrocytes has not been established. iNOS is frequently upregulated in highly active microglia that are often, but not always [84, 85], associated with mediators capable of influencing pro-inflammatory astrocytic polarization. One hypothesis is that other highly activated iNOS-immunonegative microglia modulate astrocytic phenotypes. Pro-inflammatory astrocytic polarization is likely also promoted by microglia-independent mechanisms. Pro-inflammatory astrocytes are induced by a cocktail of primarily myeloid cell-derived inflammatory factors [74, 86]. Repeated insults in the form of SRS can produce focal blood–brain barrier (BBB) dysfunction and the infiltration of peripheral immune components into the brain parenchyma [87]. Chronic BBB leakage has been observed up to one month following acute DFP intoxication [88], supporting the possibility that peripherally-derived molecules may influence astrocytic polarization even in the absence of local pro-inflammatory microglia.

### Regional differences in glial responses to acute DFP intoxication

We observed the greatest density of polarized glial cell populations in the amygdala and piriform cortex, which is consistent with our own TSPO PET data and previous reports indicating that these two brain regions are the most severely impacted by various neuropathological manifestations post-OP intoxication, including BBB dysfunction [89], neurodegeneration [15], and gliosis [15, 90, 91]. Within the amygdala-piriform area, hypertrophic polarized astrocytes were concentrated at the boundary of astrocyte-depleted zones. These astrocyte-depleted areas have been previously documented following acute DFP intoxication [90], and bare the hallmark of glial scarring [92].

In the context of acute OP intoxication, neither have these lesions been extensively characterized, nor their functional relevance explored. Moreover, despite common histological features, it is not known if these lesions develop into glial scars. If they do, there is an extensive literature detailing the cellular composition and epileptogenic implications of glial scar formation [92, 93]. The inflammatory phenotype of glial scar-associated astrocytes is not known and exploration of how these astrocytic inflammatory subpopulations influence scar physiology remains an activate area of research [94]. Regardless of astrocytic inflammatory phenotype, it is well-known in models other than acute OP intoxication that glial scar-associated astrogliosis is associated with loss of homeostatic functions, including K^+^ buffering [95] and neurotransmitter balance [96, 97], and these cellular dysfunctions have been linked to epileptogenic processes [93]. The presence of pro-inflammatory astrocytes in the amygdala and piriform cortex supports the potential for astroglial-induced chronic neurotoxicity [74]. Additional studies are needed to further explore the function of these astrocyte populations following acute OP intoxication.

Additional co-labeling experiments are required to assess the distribution of polarized microglia populations in relation to glial scarring; however, the observation of scarring and microglial lesions in serial sections suggests spatial overlap between these two processes. It has been suggested that microglia dominate the scar core in models of acute OP intoxication [90], and other scar-producing models report a high density of microglia in and around glial scars [98]. A high density of pro-inflammatory microglia in and around these amygdala-piriform lesions may influence excitability through release of inflammatory mediators, like IL-1β, that have well-defined epileptogenic effects [27]. In the current study, we did not measure expression of pro-epileptogenic molecules; however, previous work has identified upregulation of pro-inflammatory and pro-epileptogenic pathways following acute OP intoxication [12, 99–101], suggesting a role for these peri-lesion pro-inflammatory cell populations in SRS and cognitive impairment downstream of acute OP intoxication.

While the majority of research emphasizes the detrimental effects of pro-inflammatory cell populations, the presence of anti-inflammatory cell populations in our model suggests neuroprotective glial processes post-OP intoxication. Anti-inflammatory microglia are associated with phagocytic action and neurotrophic support processes [102]. Our observation of anti-inflammatory cell populations follows a body of research reporting phagocytic CD68-immunopositive microglia for months post-OP intoxication [8, 25, 91]. Similarly, anti-inflammatory astrocytes are considered neuroprotective and promote tissue repair in many conditions [102]. A hallmark of anti-inflammatory astrocytes is induction of angiogenesis [94], which has been reported in scarred areas [103]. A high density of anti-inflammatory astrocytes suggests that these cells may be beneficial to the brain microenvironment post-intoxication. Collectively, the presence of microglia and astrocyte populations with diverse inflammatory phenotypes confirms our suspicion of mixed neurotoxic and neuroprotective processes post-OP intoxication.

### Sex differences

This study was not sufficiently powered to directly compare sexes, but we did note qualitative differences in the neuroinflammatory response between males and females. While there was conservation in the spatiotemporal progression of neuroinflammatory measures between the sexes, there were differences in TSPO PET and the density of polarized glial cell populations. The magnitude of DFP-induced increases in polarized microglial cell populations was far greater in males, while astrocytic responses were similar between sexes. Across disease states, sex is known to modify immune function [104–106], and it is possible that there are innate sex differences in the extremes of pro- and anti-inflammatory glial polarization following acute OP intoxication. The apparent sex differences in neuroinflammatory response reported here parallel observations of blunted BBB dysfunction post-DFP intoxication in females relative to males despite comparable initial seizure response (unpublished data) and suggest that females may be somewhat protected against some aspects of OP-associated neuropathology.

### Therapeutic implications

Placing our findings in the context of evolving neuropathological processes [10, 107] suggests the potential for intervention to mitigate the delayed development of long-term sequelae of acute OP intoxication. The peak of DFP-induced neuronal cell death occurs within 3 DPE [59], yet there is evidence of active neurodegeneration at 60 DPE [7]. One hypothesis is that waves of neurodegeneration are differentially influenced by evolving neuroinflammatory processes. The slower onset of glial responses following the acute insult (OP-induced SE) suggests that early seizure-associated neurodegeneration, within several days of intoxication, is largely independent of immune activation. Persistent neuroinflammation, particularly the neurotoxic aspects of pro-inflammatory astrocytic activation may, however, contribute to chronic neurodegeneration observed months post-intoxication [11]. While it is not known if these chronic neuroinflammatory processes are a cause of or response to ongoing neurodegeneration, immunomodulation weeks post-intoxication should be investigated for efficacy in attenuating chronic neurodegeneration associated with acute OP intoxication.

Another neuropathologic hallmark of OP intoxication is BBB leakage within hours of intoxication that intensifies by 3 DPE and persists up to 7 [89] and 28 DPE [88]. We have observed that the first wave of DFP-induced BBB leakage is secondary to initial seizure activity (unpublished data). Similar to the progression of neurodegeneration, the timing of neuroinflammatory processes and the first wave of BBB leakage suggests that BBB leakage at early time points is likely independent of neuroinflammation. Rather, it is likely that acute BBB leakage and neurodegeneration following SE triggers early neuroinflammatory processes [108, 109]. Chronic leakage, however, correlates spatially with ongoing neuroinflammation and suggests a relationship between these two processes [107]. There is research positing a positive feedback relationship between neuroinflammation and BBB leakage [107]. In particular, the activation of astrocytes, which are critical to development and maintenance of the BBB [110], is known to interfere with many BBB functions [107, 111]. Such findings suggest that attenuation or modulation of neuroinflammatory processes may minimize chronic BBB leakage associated with acute OP intoxication.

Both the timing and localization of pro-inflammatory glial polarization suggest their potential involvement in OP-associated functional outcomes. Early pro-inflammatory polarization aligns temporally with the emergence of SRS [112] (unpublished data), while the concentration of polarized glial populations in the amygdala and piriform cortex is of particular interest given their recognition as common epileptic foci in preclinical models [113] and patients [114]. Pro-inflammatory glia are excitatory and epileptogenic in other models [115], and while the concentration of polarized glial cells in the amygdala and piriform cortex may be a response to SE-associated BBB dysfunction and neuronal cell death, the presence of these populations warrants further investigation of their potential epileptogenic role in acute OP intoxication.

Circumstantial evidence suggests the involvement of inflammatory glial cell populations in the development of cognitive deficits post-OP intoxication. The amygdala is a well-known meditator of cognition [116], and substantial research documents deficits in amygdala-mediated cognitive assessments post-OP intoxication [117, 118]. The accumulation of polarized glial populations in the amygdala supports the possible involvement of these cell populations in OP-associated cognitive impairment.

## Limitations and future directions

It is likely that our phenotypically polarized cell populations include peripheral immune cells. IBA1 immunoreactivity alone cannot differentiate between resident microglia and infiltrating monocytes and macrophages. Given the extent of damage and BBB breakdown post-OP intoxication [89], peripheral immune cells likely crossed into the brain parenchyma. While future investigation should more completely document the relative contribution of resident microglia and infiltrating peripheral immune cells, the functional overlap between microglia and peripheral monocytes and macrophages suggests that cell origin may not matter to outcome. Both central and peripheral myeloid cells can produce inflammatory molecules to influence astrocytic polarization [74, 86].

We recognize that our data are indicative of phenotypic extremism [68, 73] in that the methods employed examine glial inflammatory phenotypes through a binary lens, whereas it is known that there is a diverse continuum of phenotypic states for both microglia [119, 120] and astrocytes [121, 122]. Our findings provide a novel characterization of the spatiotemporal profile of these phenotypic extremes following acute OP intoxication, however, further studies are needed to fully investigate the presence or absence of additional glial subpopulations.

We report changes in the density of polarized cells and population-wide shifts in inflammatory phenotype across microglia and astrocytes, but we cannot speculate as to the relative contribution of these cell populations to DFP-associated pathology. Future investigations should explore the net influence of each cell population on pathology and identify if a minority of extremely polarized cells is capable of adversely impacting brain physiology.

Our use of TSPO PET imaging enabled in vivo, longitudinal assessment of neuroinflammatory processes following acute DFP intoxication. However, TSPO PET is not able to provide selectivity in assessment of specific cell populations or inflammatory phenotypes. While our data suggest that the TSPO response in the DFP rat reflects pro- and anti-inflammatory microglial and astrocytic responses, TSPO response is dependent on inflammatory stimulus and has been attributed to a variety of cell types [123–125]. With this limitation, our data do not definitively identify the origins of TSPO response in the rat following acute OP intoxication. TSPO PET, however, enabled the tracking of inflammatory response within the same animal across timepoints, not possible via histology.

Our study design did not allow for evaluating the causal relationship between glial inflammatory phenotypes and other OP-associated outcomes. We have, however, provided a foundation to identify time points for investigation of such a relationship. As previously described, the physiologic or pathophysiologic role of neuroinflammation and particular inflammatory mediators may change relative to the initial insult [11, 35]. Our findings provide the backdrop to target specific inflammatory cell populations and assess their involvement in OP-associated sequelae; for example, modulating early pro-inflammatory microglial polarization to evaluate their relative neuroprotective and/or epileptogenic role following acute OP intoxication.

## Conclusion

We provide the first comprehensive evaluation of the spatiotemporal progression of phenotypic polarization in microglia and astrocyte populations in both male and female rats following acute intoxication with the OP agent DFP. We report an environment containing mixed pro- and anti-inflammatory glial populations that change in composition over the first 28 days post-intoxication. Our data highlight the amygdala and piriform cortex areas as regions with particularly dense populations of pro- and anti-inflammatory glial cells and identify the delayed nature of astrocytic responses compared to microglial. Spatiotemporal shifts in glial phenotypes suggest therapeutic windows for anti-inflammatory interventions. Specifically, our data suggest potential benefits for a phased approach to anti-inflammatory therapies, targeting microglia earlier after exposure, within 7 days post-OP intoxication, and targeting pro-inflammatory astrocytes in more chronic stages.

## Supplementary Information

Additional file 1.

Additional file 2.

Additional file 3.

Additional file 4.

Additional file 5.

## Data Availability

Data is provided within the manuscript or supplementary information files.

## Abbreviations

AS: Atropine sulfate
BBB: Blood brain barrier
CNS: Central nervous system
DPE: Days post-exposure
DFP: Diisopropylfluorophosphate
FDR: False discovery rate
GMR: Geometric mean ratio
MDZ: Midazolam
MRI: Magnetic resonance imaging
OPs: Organophosphates
2-PAM: 2-Pralidoxime
PBS: Phosphate-buffered saline
PET: Positron emission tomography
SE: Status epilepticus
SRS: Spontaneous recurrent seizures
SUV: Standardized uptake value
SUVR: SUV ratio
SUVR_Q1_: Average SUV of the lowest quartile of voxels in the cerebellum
T-PBS: 0.03% (V/v) Triton X-100 in PBS
TSPO: 18-KDa translocator protein
Veh: Vehicle
VOI: Volume of interest
