# Supplementary material for: Shifts in the spatiotemporal profile of inflammatory phenotypes of innate immune cells in the rat brain following acute intoxication with the organophosphate diisopropylfluorophosphate
Source: J Neuroinflammation. 2024 Nov 4;21:285. doi: 10.1186/s12974-024-03272-8 (PMC11533402; doi:10.1186/s12974-024-03272-8)
Supplement: Supplementary file 5 — Additional file 5. [file 12974_2024_3272_MOESM5_ESM.docx]

**SUPPLEMENTAL MATERIAL**

**Shifts in the spatiotemporal profile of glia phenotypes in the rat brain following acute intoxication with the organophosphate diisopropylfluorophosphate**

Peter M. Andrew^1^, Jeremy A. MacMahon^1^, Pedro N. Bernardino^1^, Yi-Hua Tsai^1^, Brad A. Hobson^2^, Valerie Porter^3^, Sydney L. Huddleston^2^, Audrey S. Luo^1^, Donald A. Bruun^1^, Naomi H. Saito^4^, Danielle J. Harvey^4^, Amy Brooks-Kayal^5^, Abhijit J. Chaudhari^2,6^, Pamela J. Lein^1*^

*Corresponding author: [pjlein@ucdavis.edu](mailto:pjlein@ucdavis.edu)

^1^ Department of Molecular Biosciences, University of California, Davis, School of Veterinary Medicine, Davis, CA 95616 USA

^2^ Center for Molecular and Genomic Imaging, University of California, Davis, College of Engineering, Davis, CA 95616 USA

^3^ Department of Biomedical Engineering, University of California, Davis, College of Engineering, Davis, CA 95616, USA

^4^ Department of Public Health Sciences, University of California, Davis, School of Medicine, Davis, CA 95616 USA

^5^ Department of Neurology, University of California, Davis, School of Medicine, Sacramento, CA 95817 USA

^6^ Department of Radiology, University of California, Davis, School of Medicine, Sacramento, CA 95817 USA

**
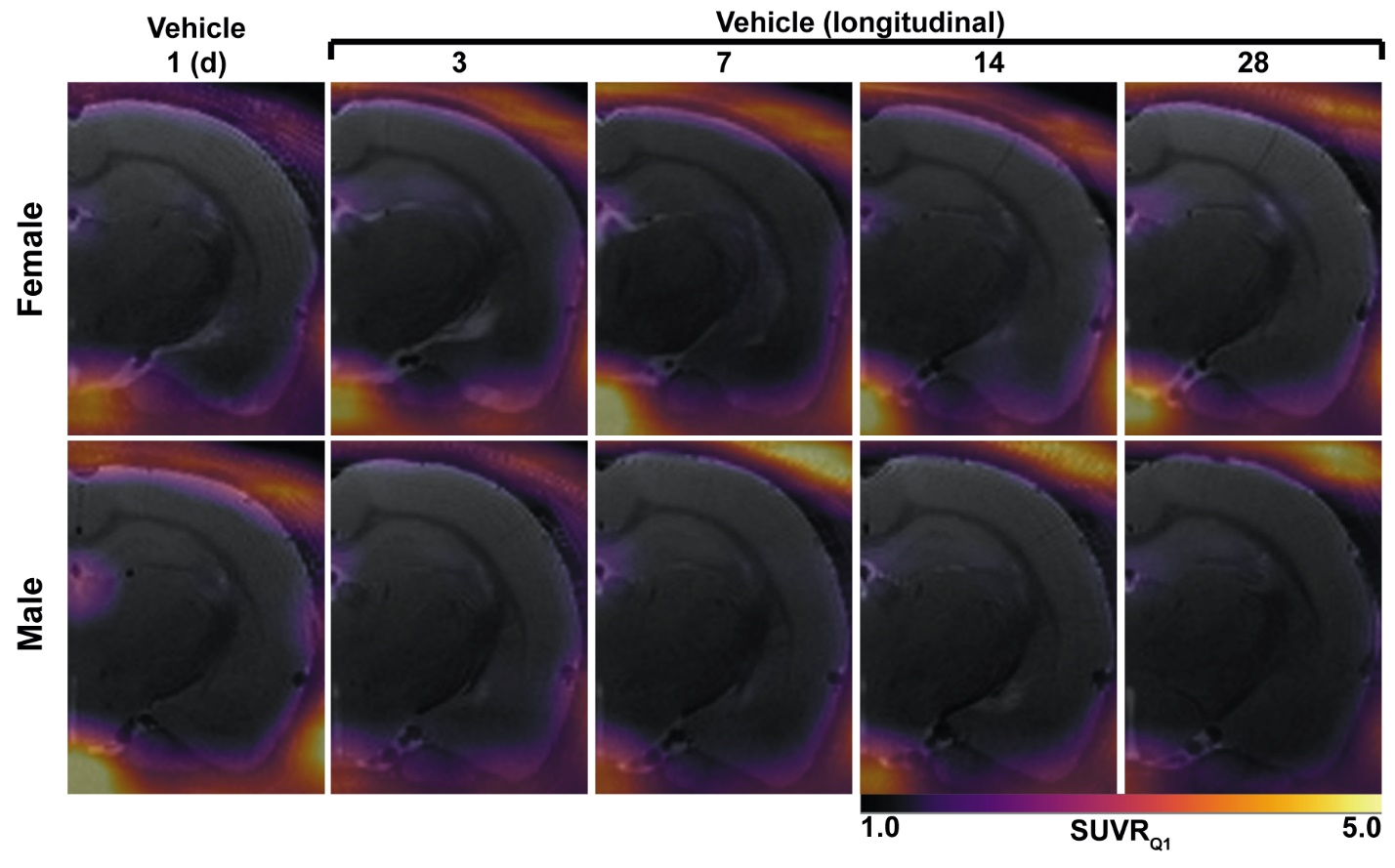
**

**Supplemental Figure 1**. (**a**) Longitudinal [18F]DPA-714 PET SUVRQ1 maps of vehicle control (VEH) animals. Data are overlaid on corresponding T2-weighted images from the same animals. The [18F]DPA714 uptake observed in the VEH animals is largely due to non-specific binding. In all scans there is minor signal penetration from outside the brain along the skull and jaw bones due to [18F] binding in bone following defluorination of the radiotracer.

**
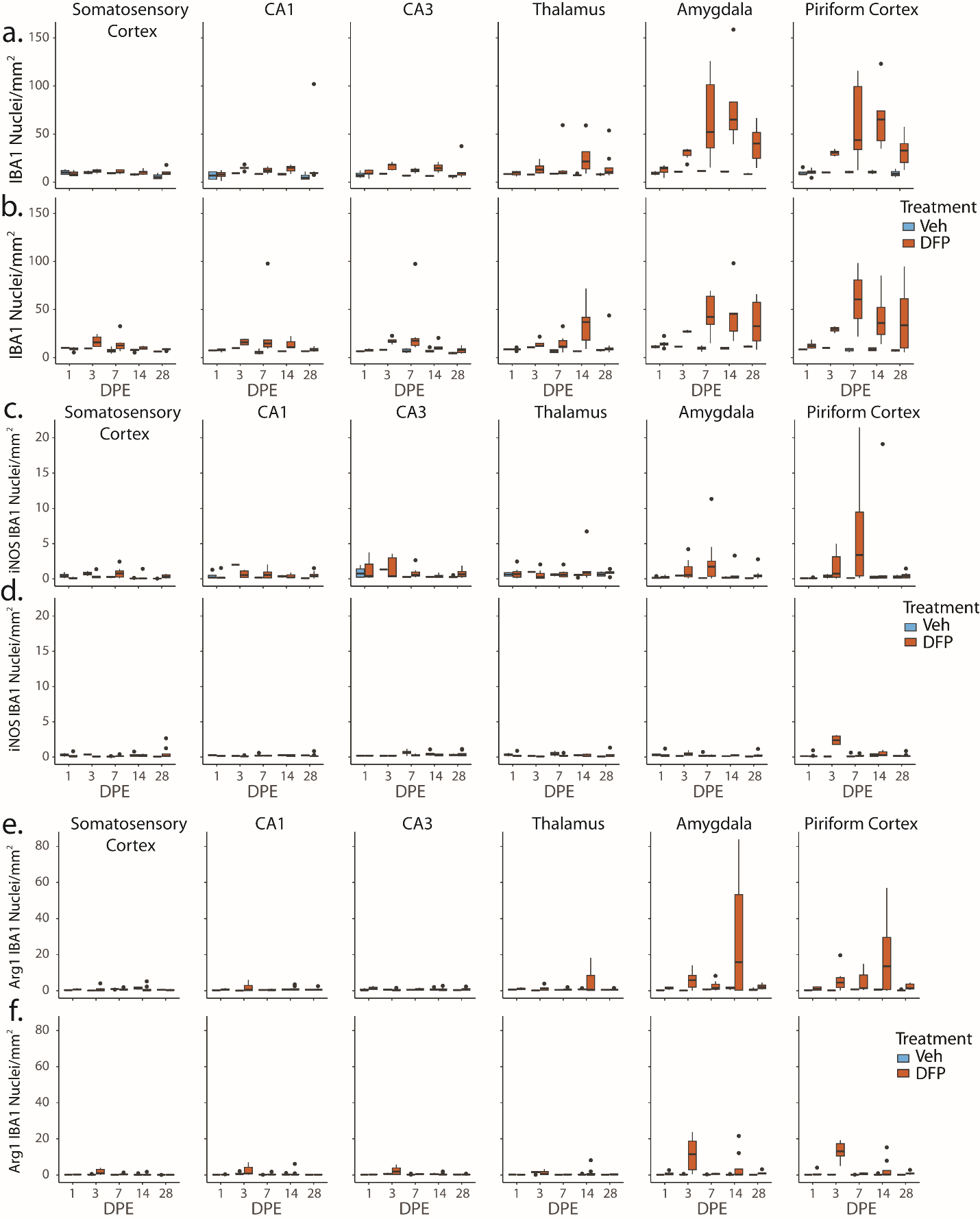
**

**Supplemental Figure 2**. (**a, b**) Density of IBA1^+^ nuclei, (**c, d**) iNOS^+^ IBA1^+^ nuclei, and (**e, f**) Arg1^+^ IBA1^+^ nuclei in different brain regions of male (**a, c, e**) and female (**b, d,** **f**) rats at 1, 3, 7, 14, and 28 d post-exposure (DPE) to Veh (blue) or DFP (red). Data are presented as boxplots in which the ends of the whiskers extend to the smallest/largest observation within 1.5 times the interquartile range of the ends of the box; ends of the box, the 25th and 75th percentiles; the horizontal line in the box, the median; and additional dots, outliers that extend beyond the whiskers (n = 2-4 Veh and 5-8 DFP per sex per timepoint).

**
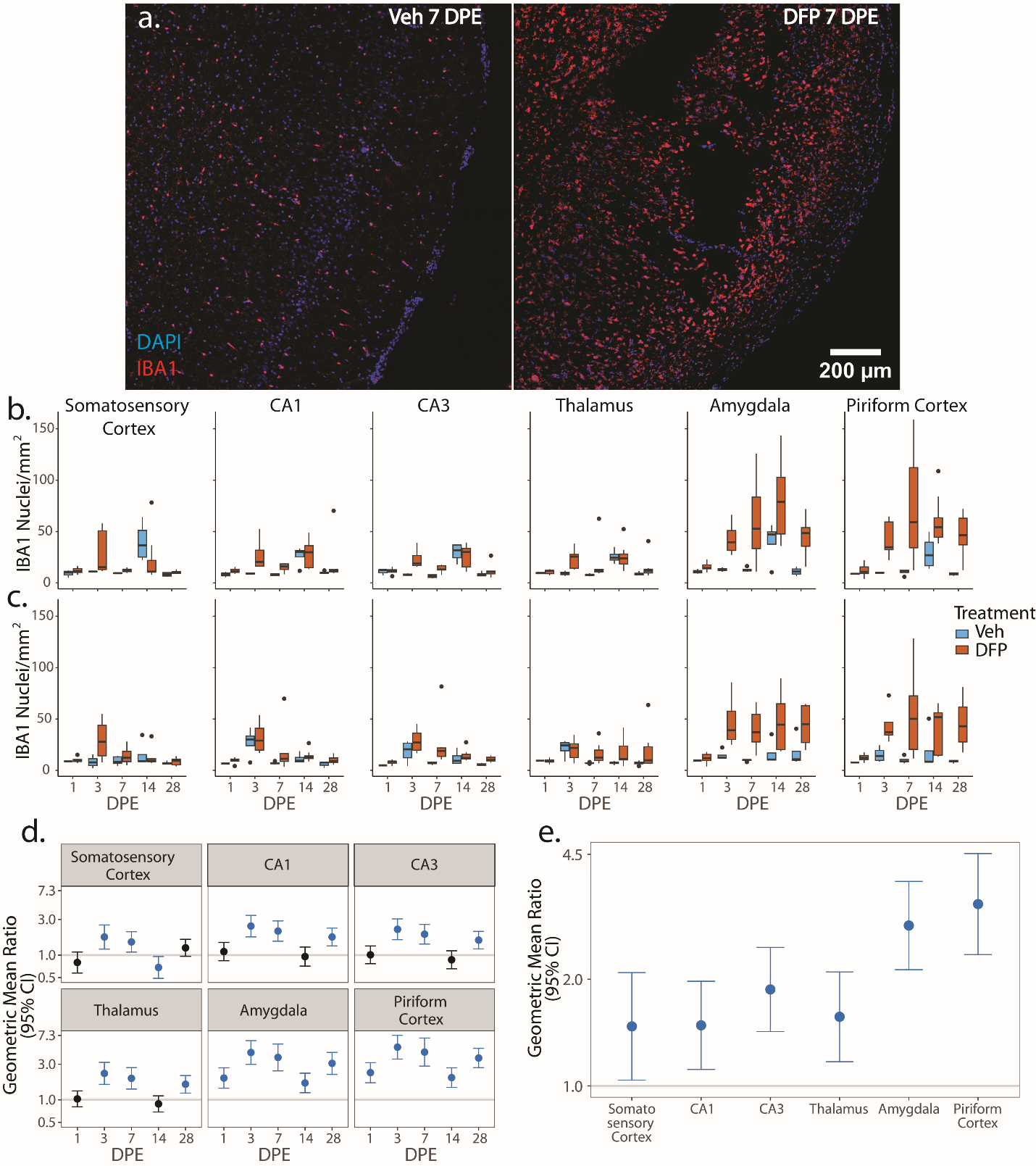
**

**Supplemental Figure 3**. **(a)** Representative photomicrographs of piriform cortex-amygdala immunostained for IBA1 (red) to identify microglia and counterstained with DAPI (blue) to identify cell nuclei. Density of IBA1^+^ nuclei in various brain regions of male (**b**) and female (**c**) rats at 1, 3, 7, 14, and 28 d post-exposure (DPE) to Veh (blue) or DFP (red). Data are presented as boxplots in which the ends of the whiskers extend to the smallest/largest observation within 1.5 times the interquartile range of the ends of the box; ends of the box indicate the 25^th^ and 75^th^ percentiles, the horizontal line in the box the median, and additional dots as outliers that extend beyond the whiskers (n = 2-4 Veh and 6-8 DFP per sex per timepoint). **(d, e)** Geometric mean ratio (GMR) (dot) of the mean IBA1^+^ nuclei density in animals intoxicated with DFP relative to Veh at 1, 3, 7, 14, and 28 DPE in various brain regions with 95% confidence intervals (bars) in males **(d)** and females **(e)**. **(e)** In females, differences in IBA1^+^ nuclei density did not vary with time post-intoxication so overall difference by brain region is displayed. The y-axis is shown as a log-scale. Confidence intervals that do not include 1 (the gray horizontal line) and are shaded blue indicate a significant difference in the density of IBA1^+^ nuclei between DFP and Veh after FDR correction.


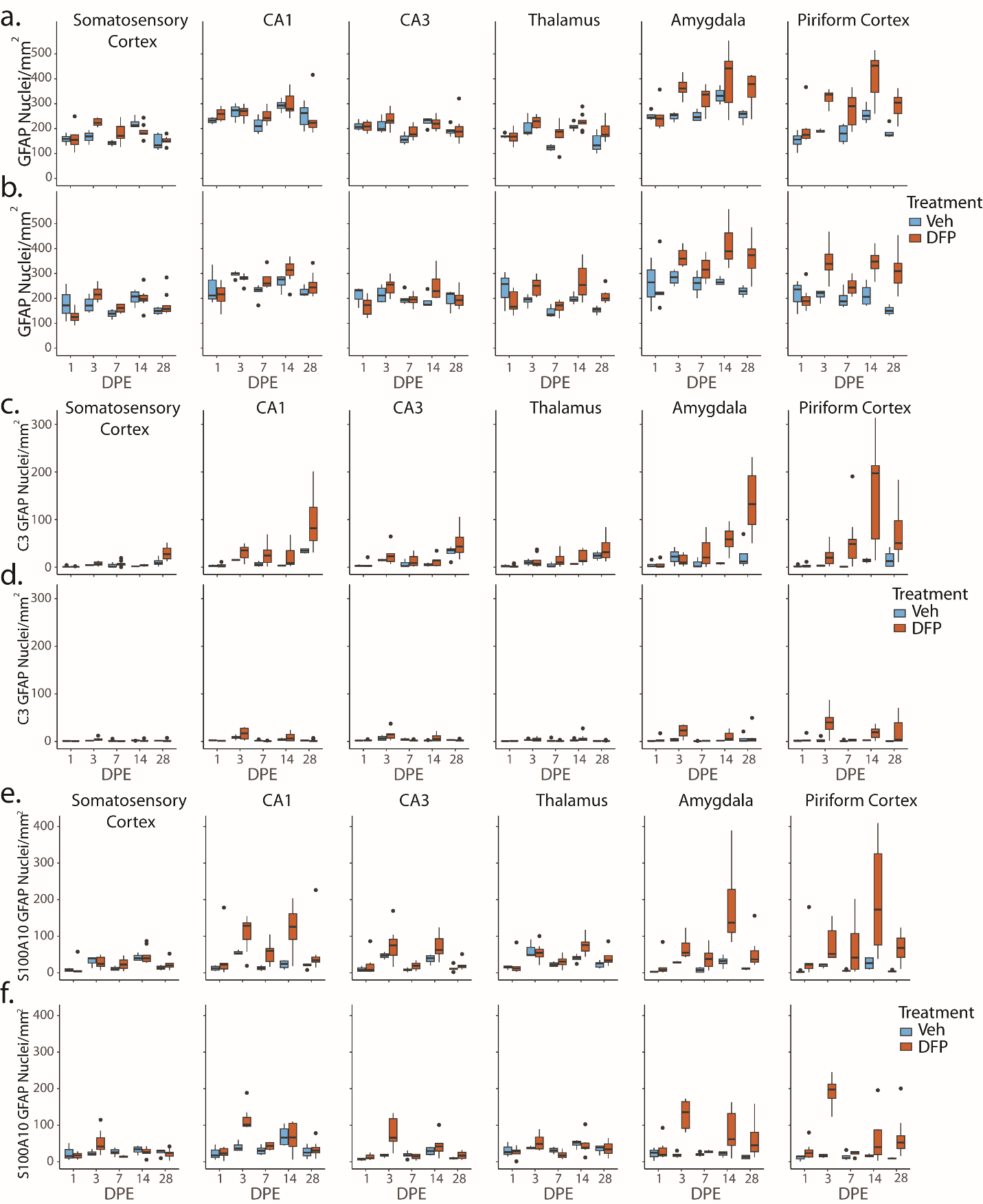

**Supplemental Figure 4**. (**a, b**) Density of GFAP^+^ nuclei, (**c, d**) C3^+^ GFAP^+^ nuclei, and (**e, f**) S100A10^+^ GFAP^+^ nuclei in various brain regions of male (**a, c, e**) and female (**b, d, f**) rats at 1, 3, 7, 14, and 28 d post-exposure (DPE) to Veh (blue) or DFP (red). Data are presented as boxplots in which the ends of the whiskers extend to the smallest/largest observation within 1.5 times the interquartile range of the ends of the box;; ends of the box , the 25^th^ and 75^th^ percentiles; the horizontal line in the box, the median; and additional dots, outliers that extend beyond the whiskers (n = 2-4 Veh and 6-8 DFP per sex per timepoint).


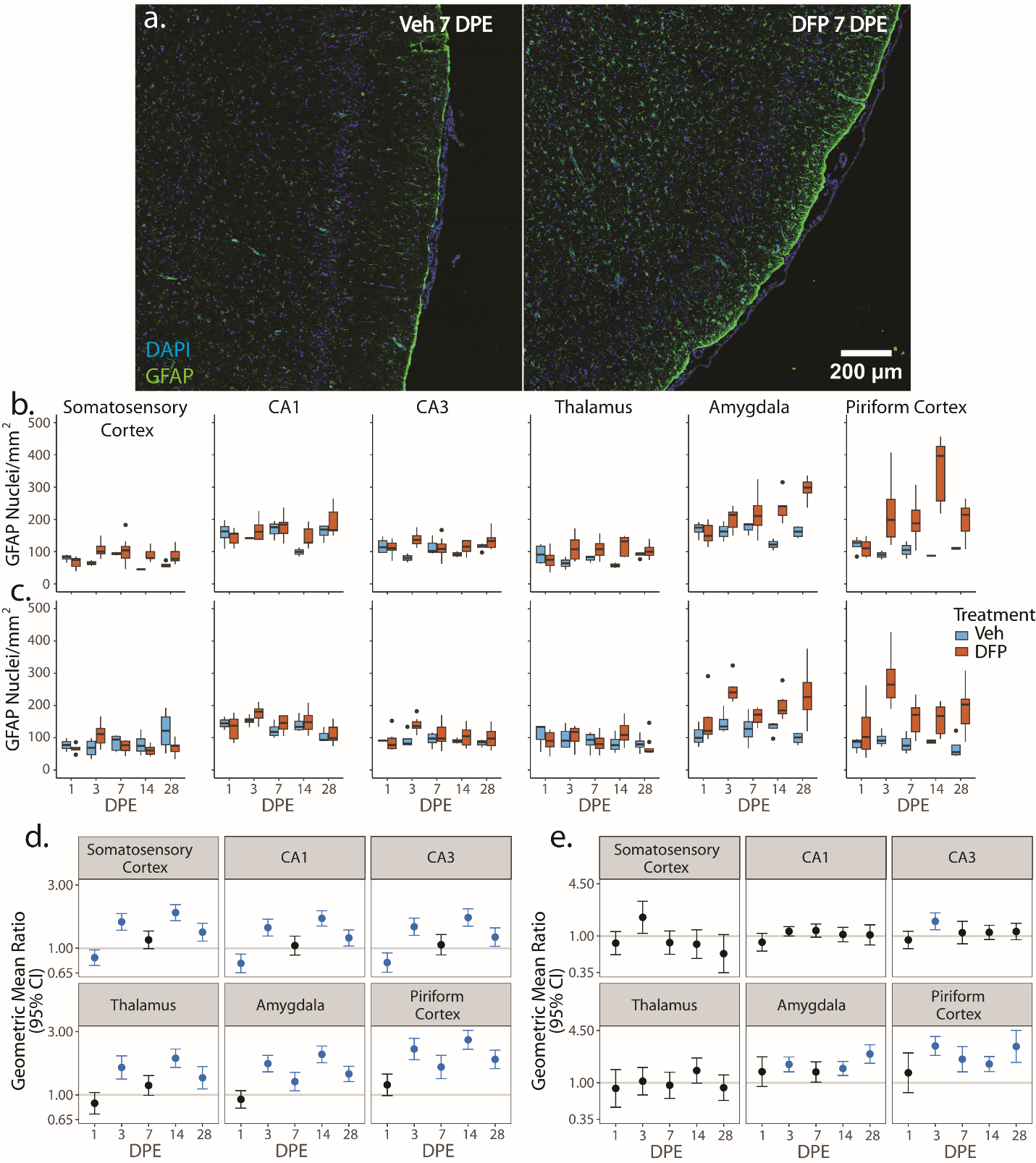


**Supplemental Figure 5**. **(a)** Representative photomicrographs of piriform cortex-amygdala immunostained for GFAP (green) to identify astrocytes and counterstained with DAPI (blue) to identify cell nuclei. Density of GFAP^+^ nuclei in various brain regions of male (**b**) and female (**c**) rats at 1, 3, 7, 14, and 28 d post-exposure (DPE) to Veh (blue) or DFP (red). Data are presented as boxplots in which the ends of the whiskers extend to the smallest/largest observation within 1.5 times the interquartile range of the ends of the box; ends of the box indicate the 25^th^ and 75^th^ percentiles, the horizontal line in the box the median, and additional dots as outliers that extend beyond the whiskers (n = 2-4 Veh and 6-8 DFP per sex per timepoint). **(d, e)** Geometric mean ratio (GMR) (dot) of the mean GFAP^+^ nuclei density in animals intoxicated with DFP relative to Veh at 1, 3, 7, 14, and 28 DPE in various brain regions with 95% confidence intervals (bars) in males **(d)** and females **(e)**. The y-axis is shown as a log-scale. Confidence intervals that do not include 1 (the gray horizontal line) and are shaded blue indicate a significant difference in the density of GFAP^+^ nuclei between DFP and Veh after FDR correction.
